# New inequalities for *p*(*n*) and $\log p(n)$

**DOI:** 10.1007/s11139-022-00653-6

**Published:** 2022-10-19

**Authors:** Koustav Banerjee, Peter Paule, Cristian-Silviu Radu, WenHuan Zeng

**Affiliations:** 1 Research Institute for Symbolic Computation (RISC), Johannes Kepler University, Altenbergerstr. 69, 4040 Linz, Austria; 2 Institute for Bioinformatics and Medical Informatics, University of Tübingen, 72076 Tübingen, Germany

**Keywords:** The partition function asymptotics, Log-concavity, Hardy–Ramanujan–Rademacher formula, primary 11P81, secondary 05A20

## Abstract

Let *p*(*n*) denote the number of partitions of *n*. A new infinite family of inequalities for *p*(*n*) is presented. This generalizes a result by William Chen et al. From this infinite family, another infinite family of inequalities for $\log p(n)$ is derived. As an application of the latter family one, for instance obtains that for $n\ge 120$,

$$\begin{aligned} p(n)^2>\Biggl (1+\frac{\pi }{\sqrt{24}n^{3/2}}-\frac{1}{n^2}\Biggr )p(n-1)p(n+1). \end{aligned}$$

## Introduction

We denote by *p*(*n*) the number of partitions of *n*. The first 50 values of *p*(*n*) starting from $n=0$ read as follows:
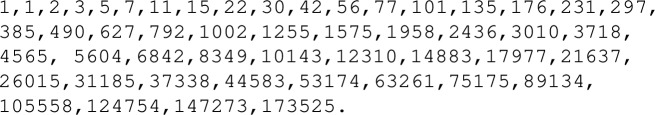


A well-known asymptotic formula for *p*(*n*) was found by Hardy and Srinivasa Ramanujan [9] in 1918 and independently by James Victor Uspensky in 1920 [15]:

1.1 $$\begin{aligned} p(n)\sim \frac{1}{4n\sqrt{3}}e^{\pi \sqrt{\frac{2n}{3}}}. \end{aligned}$$

An elementary proof of (1.1) was given by Paul Erdős [7] in 1942. At MICA 2016 (Milestones in Computer Algebra) held in Waterloo in July 2016, Zhenbing Zeng et al. [14] reported that using numerical analysis they found a better asymptotic formula^1^ for *p*(*n*) by searching for constants $C_{i,j}$ to fit the following formula:

1.2 $$\begin{aligned} \log p(n)= & {} \pi \sqrt{\frac{2}{3}}\sqrt{n}-\log n -\log (4\sqrt{3})+\frac{C_{0,-1}}{\log {n}}+\frac{C_{1,0}}{\sqrt{n}} \nonumber \\&+\frac{C_{1,-1}}{\sqrt{n}\log (n)}+\frac{C_{2,1}\log n}{n}+\frac{C_{2,0}}{n}+\cdots . \end{aligned}$$

By substituting for $n=2^{10},2^{11}, \ldots , 2^{20}$ into (1.2) they obtained,

$$\begin{aligned} C_{0,-1}=0, \quad C_{1,0}=-0.4432\ldots , \quad C_{1,-1}=0, \quad C_{2,1}=0, \quad C_{2,0}=-0.0343\ldots . \end{aligned}$$

The OEIS [12] for A000041 shows that a similarly refined asymptotic formula for *p*(*n*) was discovered by Jon E. Schoenfield in 2014, this reads

1.3 $$\begin{aligned} p(n) \sim \frac{1}{4n\sqrt{3}} e^{\pi \cdot (\frac{2n}{3} + c_0 + \frac{c_1}{\sqrt{n}} + \frac{c_2}{n} + \frac{c_3}{n\sqrt{n}} + \frac{c_4}{n^2} + \ldots )^{\frac{1}{2}}}, \end{aligned}$$

where the coefficients are approximately

$$\begin{aligned} c_0= & {} -0.230420\ldots , \quad c_1 = -0.017841\ldots , \quad c_2 = 0.005132\ldots , \\ c_3= & {} -0.001112\ldots , \quad c_4 = 0.000957\ldots , \end{aligned}$$

Later Vaclav Kotesovec according to OEIS [12] for A000041 got the precise value of $c_0, c_1,\ldots , c_4$ as follows:

$$\begin{aligned} c_0= & {} -\frac{1}{36} - \frac{2}{\pi ^2}, \quad c_1 = \frac{1}{6\sqrt{6}\pi } - \frac{\sqrt{6}}{2\pi ^3}, \quad c_2 = \frac{1}{2\pi ^4},\\ c_3= & {} - \frac{5}{16\sqrt{6}\pi ^3}+\frac{3\sqrt{6}}{8\pi ^5} , \quad c_4 = \frac{1}{576\pi ^2} - \frac{1}{24\pi ^4} + \frac{93}{80\pi ^6}. \end{aligned}$$

To the best of our knowledge, the details of the methods of Schoenfield and Kotesovec have not yet been published.

In this article, using symbolic-numeric computation, we present our method to derive (1.2) together with a closed form formula for the $C_{i,j}$ in (1.2). Namely we show that

$$\begin{aligned} \log p(n)\sim \pi \sqrt{\frac{2n}{3}}-\log n-\log 4\sqrt{3}+\sum _{u=1}^{\infty }\frac{g_u}{\sqrt{n}^u}, \end{aligned}$$

where the $g_u$ are as in Definition 5.1. By $\sim$ in the above expression we mean that for each $N\ge 1$

$$\begin{aligned} \log p(n)=\pi \sqrt{\frac{2n}{3}}-\log n-\log 4\sqrt{3}+\sum _{u=1}^{N-1}\frac{g_u}{\sqrt{n}^u}=O_N(n^{-N/2}). \end{aligned}$$

In particular $C_{i,j}=0$, if $j\ne 0$, and $C_{i,0}=g_i$, otherwise. This result is obtained as a consequence of an infinite family of inequalities for $\log p(n)$, Theorem 6.6 (main theorem). We also apply our method to conjecture an analogous formula to (1.2) for *a*(*n*), the cubic partitions of *n*, with *a*(*n*) given by

1.4 $$\begin{aligned} \sum _{n=0}^{\infty }a(n)q^n=\prod _{n=1}^{\infty }\frac{1}{(1-q^n)(1-q^{2n})}. \end{aligned}$$

In the OEIS, this sequence is registered as A002513. The first 50 values of *a*(*n*), $n\ge 0$, are
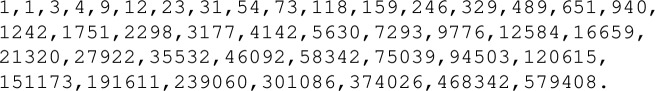


This sequence appears in a letter from Richard Guy to Morris Newman [8]. In [4], William Chen and Bernard Lin proved that the sequence *a*(*n*) satisfies several congruence properties. For example, $a(3n+2)\equiv 0\pmod {3}$, $a(25n+22) \equiv 0\pmod {5}$. An asymptotic formula for *a*(*n*) was obtained by Kotesovec [10] in 2015 as follows:

1.5 $$\begin{aligned} a(n)\sim \frac{e^{\pi \sqrt{n}}}{8n^{5/4}}. \end{aligned}$$

In [16] the fourth author investigated the combinatorial properties of the sequence *a*(*n*) by using Maple.

We summarize some of our main results:

### Theorem 1.1

For the usual partition function *p*(*n*) we have

1.6 $$\begin{aligned} \log p(n) \sim \pi \sqrt{\frac{2n}{ 3}} - \log n - \log 4\sqrt{3} - \frac{0.44\ldots }{\sqrt{n} }, \ n\rightarrow \infty . \end{aligned}$$

The proof of this theorem will be given in Sect. 6.

### Conjecture 1.2

For the cubic partitions *a*(*n*) we have

1.7 $$\begin{aligned} \log a(n) \sim \pi \sqrt{n} - \frac{5}{4}\log n - \log 8 - \frac{0.79\ldots }{\sqrt{n} }, \ n \rightarrow \infty . \end{aligned}$$

### Theorem 1.3

For the partition numbers *p*(*n*) we have the inequalities

$$\begin{aligned} \frac{e^{\pi \sqrt{\frac{2n}{3}}}}{4\sqrt{3}n} \Bigl (1 - \frac{1}{2\sqrt{n}}\Bigr )< p(n) < \frac{e^{\pi \sqrt{\frac{2n}{3}}}}{4\sqrt{3}n} \Bigl (1 - \frac{1}{3\sqrt{n}}\Bigr ), \ n\ge 1. \end{aligned}$$

The proof of this is given in Sect. 3.

This paper is organized as follows. In Sect. 2 we present the methods used in the mathematical experiments that led us Theorem 1.1 and Conjecture 1.2. In Sect. 3 we prove Theorem 1.3 by adapting methods used by Chen et al. to fit our purpose. In Sect. 4 we generalize an inequality by Chen et al. by extending it to an infinite family of inequalities for *p*(*n*). In Sect. 5 we introduce preparatory results required to prove Theorem 6.6. In Sect. 6 we prove our main result, Theorem 6.6, by using the main result from Sect. 4, Theorem 4.4. This gives an infinite family of inequalities for $\log p(n)$. Finally in Sect. 7 we give an application of the results in Sect. 5 which extends DeSalvo’s and Pak’s log concavity theorem for *p*(*n*). In Sect. 1 (the Appendix) we give additional information on the method used to discover the asymptotic formulas. We remark explicitly that to finalize the proof of Theorem 6.6, we use the Cylindrical Algebraic Decomposition in Mathematica; the details of this are also put to Sect. 1.

## Mathematical experiments for better asymptotics for *a*(*n*) and *p*(*n*)

Before proving our theorems, in this section we briefly describe the experimental mathematics which led us to their discovery. Our strategy is as follows. If we have sufficiently many instances of a given sequence, how can we find an asymptotic formula for this sequence? Take the cubic partitions *a*(*n*) and the partition numbers *p*(*n*) as examples.

We have

$$\begin{aligned} p(10)= & {} 42,\ldots ,p(100) = \text{190569292, }\ldots ,\\ p\text{(1000) }= & {} \text{24061467864032622473692149727991, } \\ a(10)= & {} 118,\ldots ,a(100) = \text{16088094127 }, \ldots ,\\ a(1000)= & {} \text{302978131076521633719614157876165279276. } \\ \end{aligned}$$

**Fig. 1:**
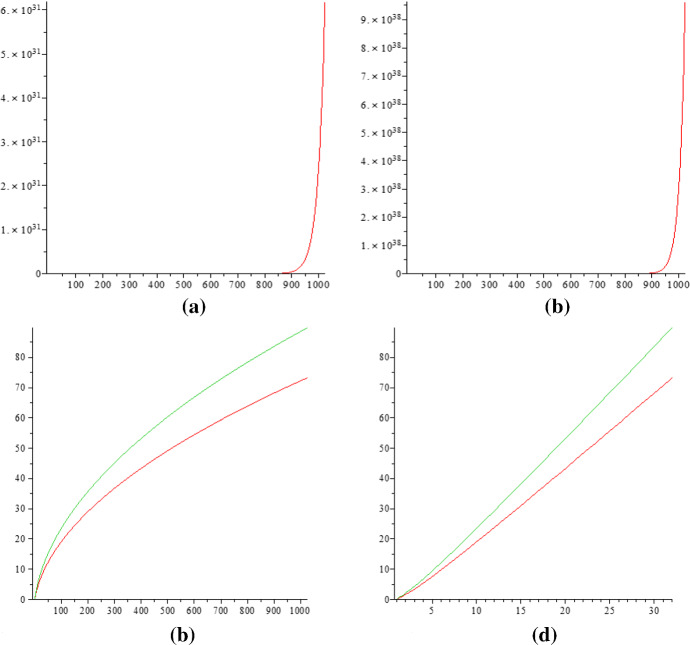
In (**a**) *p*(*n*) is plotted and in (**b**) *a*(*n*) is plotted. In (**c**) the upper curve is ${\{}(n,\log a(n))|1 \le n \le 1000\}$, and the lower curve is ${\{}(n,\log p(n))|1 \le n \le 1000\}$. The two curves are like the parabola $y=\sqrt{x}$. In (**d**) the two lines are for ${\{}(\sqrt{n} ,\log a(n))| 1 \le n \le 1000\}$ (upper) and ${\{}(\sqrt{n} ,\log p(n))|1 \le n \le 1000\}$ (lower)

A plot of the two curves through the points (*n*, *a*(*n*)), resp. (*n*, *p*(*n*)), for $n\in \{1,\ldots ,1000\}$ is shown in the Fig. 1a and  b. According to the Hardy–Ramanujan Theorem 1.1 and the asymptotic formula of Kotesovec (1.5), the curves are increasing with “sub-exponential” speeds. Thus, we may plot two curves using data points $(n, \log a(n))$ and $(n, \log p(n))$ as shown in Fig. 1c. One observes that the new curves look like parabolas $y=\sqrt{x}$. This is also very natural in view of

2.1 $$\begin{aligned} \log p(n)\sim & {} \sqrt{\frac{2}{3}} \pi \cdot \sqrt{n} - \log n - \log 4\sqrt{3} ,\nonumber \\ \log a(n)\sim & {} \pi \cdot \sqrt{n} - \frac{5}{4} \cdot \log n - \log 8. \end{aligned}$$

So if we modify further with $(\sqrt{n},\log a(n))$ and $(\sqrt{n},\log p(n))$ to plot the curves, we get two almost-straight lines as shown in the Fig. 1d.

This provides the starting point for finding the improved asymptotic formulas (1.6) for *p*(*n*) and (1.7) for *a*(*n*) from their data sets. We restrict our description to the latter case. Motivated by (2.1), we compute the differences of $\log a(n)$ with the estimation values $a_e(n):= \frac{e^{\pi \sqrt{n}}}{ 8n^{5 / 4}}$:

$$\begin{aligned} \Delta (n) := \log a_e(n) - \log a(n) = \pi \sqrt{n} - \frac{5}{4}\log n - \log 8 - \log a(n). \end{aligned}$$

**Fig. 2:**
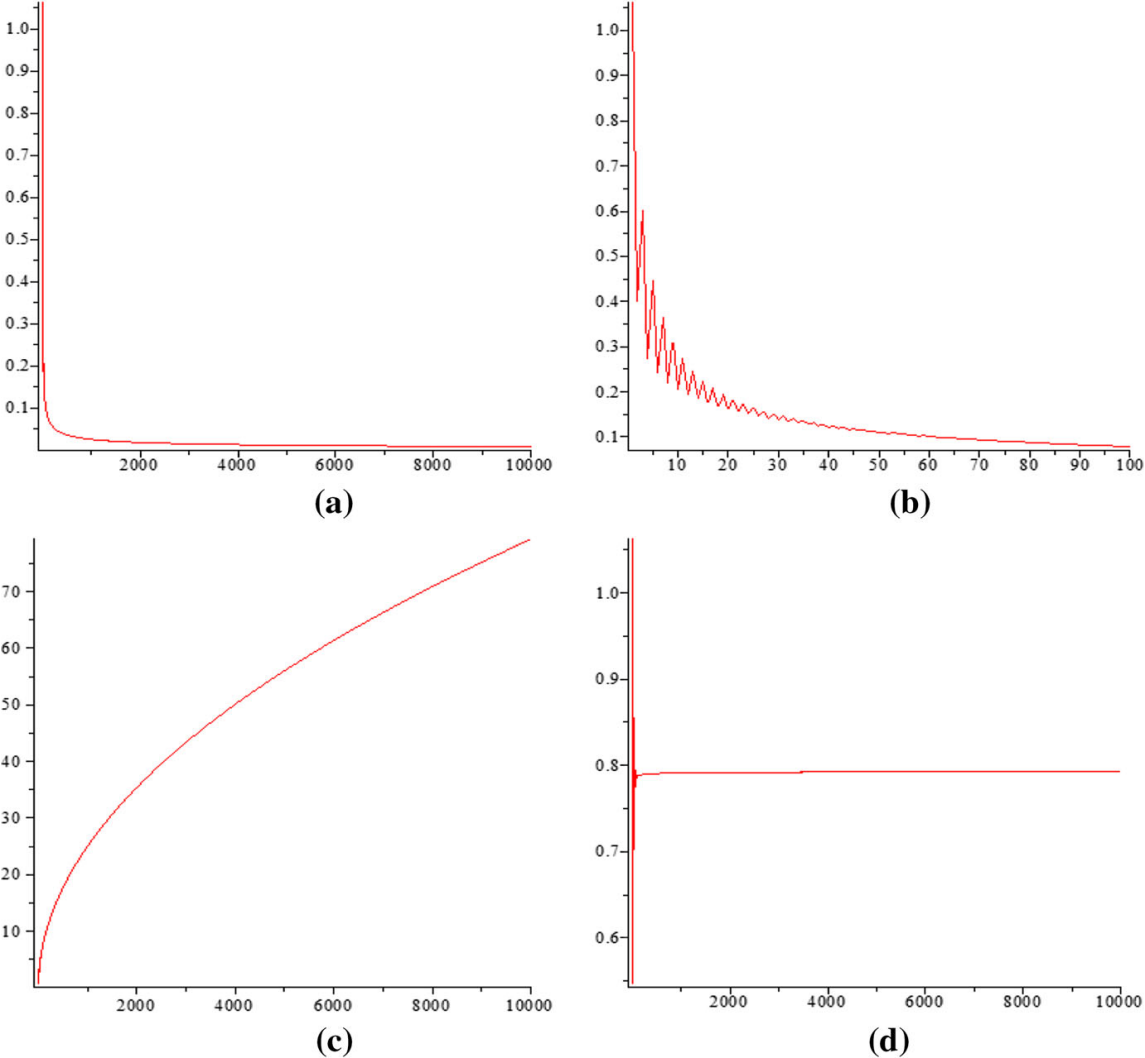
The curve in (**a**) is for $(n,\Delta (n))$ where $1\le n \le 10,000$, **b** is for $(n,\Delta (n))$ where $1\le n\le 100$. The curve in (**c**) is for $(n,n\cdot \Delta (n))$, and **d** is for $(n,\sqrt{n}\cdot \Delta (n))$ where $1\le n\le 10,000$

Then we can plot curves from the data points $(n,\Delta (n))$ in Fig. 2a and b, and $(n, n\cdot \Delta (n))$ and $(n, \sqrt{n}\cdot \Delta (n))$ in Fig. 2c and d, in order to confirm the next dominant term approximately. We can see in Fig. 2d that after multiplying $\Delta (n)$ by $\sqrt{n}$ the curve is almost constant, confirming that the next term is $\frac{C}{\sqrt{n}}$. Also multiplying $\Delta (n)$ by *n*, in Fig. 2c we see that the behavior is like $\sqrt{n}$ as expected. By using least square regression on the original data set (*n*, *a*(*n*)) for $1\le n\le 10000$, we aimed at finding the best constant *C* that minimizes^2^

$$\begin{aligned} -\log a(n) + \alpha \cdot \sqrt{n} - \beta \cdot \log n - \log \gamma +\frac{C}{\sqrt{n}}, \end{aligned}$$

where we fixed $\alpha =\pi , \beta =5/4, \gamma =8$ according to (1.5). As a result, we obtained that $C\approx 0.7925$.

In the Appendix, Sect. 1, we explain that the constants $\alpha , \beta , \gamma$ can also be found via regression analysis with Maple instead of getting them from (1.5) directly.

## Proof of Theorem 1.3

We separate the proof into two lemmas. The first lemma is the upper bound for *p*(*n*) and second lemma is the lower bound. In order to prove these lemmas we will state several facts which are routine to prove.

### Lemma 3.1

For all $n\ge 1$, we have

$$\begin{aligned} p(n) < \frac{e^{\pi \sqrt{\frac{2n}{3}}}}{4\sqrt{3}n} \Bigl (1 - \frac{1}{3\sqrt{n}}\Bigr ). \end{aligned}$$

### Proof

By [1, (2.7)–(2.8)] and with $A_k(n)$ and *R*(*n*, *N*)^3^ as defined there, we have,

$$\begin{aligned} p(n)&=\frac{\sqrt{12}}{24n-1}\sum _{k=1}^{N}\frac{A_{k}(n)}{\sqrt{k}}\Bigl [\Bigl (1-\frac{k}{\mu (n)}\Bigr )e^{\frac{\mu (n)}{k}}+\Bigl (1+\frac{k}{\mu (n)}\Bigr )e^{-\frac{\mu (n)}{k}}\Bigr ]\\&\quad +R(n,N), \ n\ge 1, \end{aligned}$$

where

$$\begin{aligned} \mu (n):=\frac{\pi }{6}\sqrt{24n-1}. \end{aligned}$$

We will exploit the case $N=2$ together with $A_{1}(n)=1$ and $A_{2}(n)=(-1)^{n}$ for any positive integer *n*. For $N\ge 1$, Lehmer [11, (4.14), p. 294] gave the following error bound:

3.1 $$\begin{aligned} | R(n,N)| < \frac{\pi ^{2}N^{-2/3}}{\sqrt{3}}\Bigl [\Bigl (\frac{N}{\mu (n)}\Bigr )^{3}\sinh \frac{\mu (n)}{N}+\frac{1}{6}-\Bigl (\frac{N}{\mu (n)}\Bigr )^{2}\Bigr ], \ n\ge 1, \end{aligned}$$

and for $N=2$ (cf. [1, (2.9)-(2.10)]):

3.2 $$\begin{aligned} p(n) = \frac{\sqrt{12}e^{\mu (n)}}{24n-1}\Bigl (1-\frac{1}{\mu (n)}+T_1(n)\Bigr ), \ n\ge 1, \end{aligned}$$

where

$$\begin{aligned} \begin{aligned} T_1(n)&:= \frac{(-1)^{n}}{\sqrt{2}}\Bigl (\Bigl (1-\frac{2}{\mu (n)}\Bigr )e^{-\frac{\mu (n)}{2}}+\Bigl (1+\frac{2}{\mu (n)}\Bigr )e^{-\frac{3\mu (n)}{2}}\Bigr )\\&+\Bigl (1+\frac{1}{\mu (n)}\Bigr )e^{-2\mu (n)}+\frac{(24n-1)R(n,2)}{\sqrt{12}e^{\mu (n)}}. \end{aligned} \end{aligned}$$

We first estimate the absolute value of $T_1(n)$; for convenience we denote subexpressions by $a_1$, $b_1$, $c_1$, and $d_1$:

$$\begin{aligned} \begin{aligned} |T_1(n)| \le&\underbrace{\frac{1}{\sqrt{2}}\Bigl (1-\frac{2}{\mu (n)}\Bigr ) e^{-\frac{\mu (n)}{2}}}_{=:a_1}+\underbrace{\frac{1}{\sqrt{2}}\Bigl (1+\frac{2}{\mu (n)}\Bigr )e^{-\frac{3\mu (n)}{2}}}_{=:b_1}\\&+\underbrace{\Bigl (1+\frac{1}{\mu (n)}\Bigr )e^{-2\mu (n)}}_{=:c_1}+\underbrace{ \Bigl |\frac{(24n-1)R(n,2)}{\sqrt{12}e^{\mu (n)}}\Bigr |}_{=:d_1}. \end{aligned} \end{aligned}$$

The following facts are easily verified.

### Fact A

For all $$n \ge 1$$, $$a_{1} < e^{-\frac{\mu (n)}{2}}$$.

### Fact B

For all $$n \ge 1$$, $$b_{1} < e^{-\frac{\mu (n)}{2}}$$.

### Fact C

For all $$n \ge 1$$, $$c_{1} < e^{-\frac{\mu (n)}{2}}$$.

Now,

$$\begin{aligned} \begin{aligned} d_1&= \frac{36}{\pi ^{2}\sqrt{12}}\frac{\mu (n)^{2}}{e^{\mu (n)}}| R(n,2)|\\&< \frac{\mu (n)^{2}e^{-\mu (n)}}{2^{2/3}}+\frac{12 \root 3 \of {2}e^{-\frac{\mu (n)}{2}}}{\mu (n)}-\frac{12 \root 3 \of {2}e^{-\frac{3\mu (n)}{2}}}{\mu (n)}-12\root 3 \of {2}e^{-\mu (n)}  (\text { by } \mathrm{(3.1)})\\&< \underbrace{\frac{\mu (n)^{2}e^{-\mu (n)}}{2^{2/3}}}_{=:d_1^{*}}+\underbrace{\frac{12 \root 3 \of {2}e^{-\frac{\mu (n)}{2}}}{\mu (n)}}_{=:d_2^{*}}. \end{aligned} \end{aligned}$$

### Fact D

For all $$n \ge 7$$, $$d^{*}_{1} < e^{-\frac{\mu (n)}{2}}$$.

### Fact E

For all $$n \ge 35$$, $$d^{*}_{2} < e^{-\frac{\mu (n)}{2}}$$.

By Fact D and Fact E, we have

### Fact F

$$d_{1} = d^{*}_{1}+ d^{*}_{2}< 2e^{-\frac{\mu (n)}{2}}$$ for all $$n \ge 35$$.

Now, by Fact A, B, C, and Fact F we conclude that for all $n \ge 35$,

3.3 $$\begin{aligned} | T_1(n)| \le a_{1}+b_{1}+c_{1}+d_{1} < 5e^{-\frac{\mu (n)}{2}}. \end{aligned}$$

By (3.3), we have for all $n \ge 35$ that

3.4 $$\begin{aligned} 1-\frac{1}{\mu (n)}- 5e^{-\frac{\mu (n)}{2}}<1-\frac{1}{\mu (n)}+T_1(n) < 1-\frac{1}{\mu (n)}+5e^{-\frac{\mu (n)}{2}}. \end{aligned}$$

### Fact G

For all $$n \ge 3$$, $$1-\frac{1}{\mu (n)}- 5e^{-\frac{\mu (n)}{2}} > 0$$.

Therefore from (3.2), (3.4), and Fact G, we have for all $n \ge 35$,

3.5 $$\begin{aligned} p(n)= & {} \frac{\sqrt{12}e^{\mu (n)}}{24n-1}\Bigl (1-\frac{1}{\mu (n)}+T_1(n)\Bigr )\nonumber \\< & {} \underbrace{\frac{\sqrt{12}e^{\mu (n)}}{24n-1}}_{=:e_1}\underbrace{\Bigl ( 1-\frac{1}{\mu (n)}+5e^{-\frac{\mu (n)}{2}}\Bigr )}_{=:f_1}. \end{aligned}$$

### Fact H

$$f_1 < 1-\frac{1}{3\sqrt{n}}$$ for all $$n \ge 23$$.

### Fact I

$$e_1 < \frac{1}{4n\sqrt{3}}e^{\pi \sqrt{\frac{2n}{3}}}$$ for all $$n \ge 1$$.

Therefore by Facts H, I, and (3.5), we have for all $n \ge 35$,

$$\begin{aligned} p(n) < \frac{1}{4n\sqrt{3}}e^{\pi \sqrt{\frac{2n}{3}}}\Bigl (1-\frac{1}{3\sqrt{n}}\Bigr ). \end{aligned}$$

This completes the proof of the stated upper bound in Lemma 3.1. $\square$

### Lemma 3.2

For all $$n \ge 1$$,

3.6 $$\begin{aligned} \frac{1}{4n\sqrt{3}}e^{\pi \sqrt{\frac{2n}{3}}}\Bigl (1 - \frac{1}{2\sqrt{n}}\Bigr ) < p(n).\end{aligned}$$

### Proof

In the proof of [6, Prop 2.4], it is noted that for all $n \ge 1$,

$$\begin{aligned} p(n) > T_2(n) \Bigl (1 - \frac{| R(n)|}{T_2(n)}\Bigr ), \end{aligned}$$

where

$$\begin{aligned} T_2(n) := \frac{\sqrt{12}}{24n-1}\Bigl [\Bigl (1-\frac{1}{\mu (n)}\Bigr )e^{\mu (n)} + \frac{(-1)^{n}}{\sqrt{2}}e^{\frac{\mu (n)}{2}}\Bigr ] \end{aligned}$$

and *R*(*n*) is as in [6, (7)].

From the definition of $T_2(n)$ one verifies:

### Fact J

$$T_2(n) > 0$$ for all $$n \ge 1$$.

The following bound holds for |*R*(*n*)| (see [6, (13)]),

$$\begin{aligned} 0< \frac{| R(n)|}{T_2(n)} < e^{-\frac{\pi }{10}\sqrt{\frac{2n}{3}}}, \ n\ge 2. \end{aligned}$$

Hence by Fact J,

3.7 $$\begin{aligned} T_2(n) \Bigl (1 - \frac{| R(n)|}{T_2(n)}\Bigr ) > T_2(n) \Bigl (1 - e^{-\frac{\pi }{10}\sqrt{\frac{2n}{3}}}\Bigr ), \ n\ge 2. \end{aligned}$$

Plugging the definition of $T_2(n)$ into (3.7) gives for $n\ge 2$,

$$\begin{aligned} \begin{aligned} p(n)&> \frac{\sqrt{12}}{24n-1}\Bigl [\Bigl (\underbrace{1-\frac{1}{\mu (n)}}_{=:a_2}\Bigr ) e^{\mu (n)} + \frac{(-1)^{n}}{\sqrt{2}}e^{\frac{\mu (n)}{2}}\Bigr ]\underbrace{(1 - e^{-\frac{\pi }{10}\sqrt{\frac{2n}{3}}})}_{=:d_2}\\&> \frac{\sqrt{12}}{24n}e^{\pi \sqrt{\frac{2n}{3}}}\Bigl [a_2\times \underbrace{e^{\mu (n) - \frac{\pi }{6}\sqrt{24n}}}_{=:b_2} + \underbrace{\frac{(-1)^{n}}{\sqrt{2}}e^{\frac{\mu (n)}{2} - \frac{\pi }{6}\sqrt{24n}}}_{=:c_2}\Bigr ]\times d_2\\&= \frac{1}{4\sqrt{3}n}e^{\pi \sqrt{\frac{2n}{3}}} (a_{2}b_{2}+c_{2})d_{2}. \end{aligned} \end{aligned}$$

We now bound $a_2$, $b_2$, $c_2$, and $d_2$:

### Fact K

$$a_2> 1 -\frac{2}{5\sqrt{n}} > 0$$ for all $$n \ge 1$$.

### Fact L

$$b_2> 1 - \frac{2}{37\sqrt{n}} > 0$$ for all $$n \ge 1$$.

### Fact M

$$c_2 > - \frac{1}{225\sqrt{n}}$$ for all $$n \ge 29$$.

### Fact N

$$d_2> 1 - \frac{1}{25\sqrt{n}}>0$$ for all $$n \ge 631$$.

By Facts K, L, and M, we have,

### Fact O

$$a_{2}b_{2}+c_{2}> (1 -\frac{2}{5\sqrt{n}})(1 - \frac{2}{37\sqrt{n}})- \frac{1}{225\sqrt{n}} > 0$$ for all $$n \ge 1$$.

From Facts O and N we have for all $n \ge 631$,

$$\begin{aligned} (a_{2}b_{2}+c_{2})d_{2} > \underbrace{\Bigl [\Bigl (1 -\frac{2}{5\sqrt{n}}\Bigr )\Bigl (1 - \frac{2}{37\sqrt{n}}\Bigr )-\frac{1}{225\sqrt{n}}\Bigr ]\Bigl (1 - \frac{1}{25\sqrt{n}}\Bigr )}_{=:I(n)}. \end{aligned}$$

### Fact P

$$I(n)> 1 - \frac{1}{2\sqrt{n}} > 0$$,   for all $$n \ge 1$$.

From all the above facts we can conclude that (3.6) holds for all $n \ge 631$. Using Mathematica we checked that (3.6) also holds for all $1 \le n \le 630$. This concludes the proof of Lemma 3.2. $\square$

Finally, combining Lemmas 3.1 and 3.2, we have Theorem 1.3.

## A generalization of a result by Chen, Jia, and Wang

In this section, we have again that $\mu (n)=\frac{\pi }{6}\sqrt{24n-1}$; this should not be confused with the real variable $\mu$ which we will use below. Eventually, we will set the real variable $\mu$ equal to $\mu (n)$. The main goal of this section is to generalize [1, Lem. 2.2] which says that for $n\ge 1206$, we have

$$\begin{aligned} \frac{\sqrt{12}e^{\mu (n)}}{24n-1}\Bigl (1-\frac{1}{\mu (n)}-\frac{1}{\mu (n)^{10}}\Bigr )<p(n)<\frac{\sqrt{12}e^{\mu (n)}}{24n-1}\Bigl (1-\frac{1}{\mu (n)}+\frac{1}{\mu (n)^{10}}\Bigr ). \end{aligned}$$

Our improvement is Theorem 4.4 where we replace the 10 in this formula by *k* and the 1206 by a parametrized bound *g*(*k*). In order to achieve this, for a fixed *k* one needs to find an explicit constant $\nu (k)\in {\mathbb {R}}$ such that $\frac{1}{6}e^{\mu /2}>\mu ^k$ for all $\mu \in {\mathbb {R}}$ with $\mu >\nu (k)$. One can show that

$$\begin{aligned} {\tilde{\nu }}(k):=\min \Bigl \{h\in {\mathbb {R}} \big | \forall _{\mu \in {\mathbb {R}}}\Bigl (\mu>h \Rightarrow \frac{1}{6}e^{\mu /2}>\mu ^k\Bigr )\Bigr \} \end{aligned}$$

satisfies $\frac{1}{6}e^{{\tilde{\nu }}(k)/2}={\tilde{\nu }}(k)^k$. Theorem 4.4 is crucial for proving our main result, Theorem 6.6, presented in the next section. In Lemma 4.1 we find such a constant $\nu (k)$ for all $k\ge 7$. In Lemma 4.2 we find a lower bound on ${\tilde{\nu }}(k)$. In this way, we see that what is delivered by Lemma 4.1 is best possible in the sense that our $\nu (k)$ from Lemma 4.1 and the minimal possible ${\tilde{\nu }}(k)$ satisfies $|\nu (k)-{\tilde{\nu }}(k)|<\frac{3k\log \log k}{\log k}$ for all $k\ge 7$.

### Lemma 4.1

For $$k\ge 7$$ let

$$\begin{aligned} \nu (k):=2\log 6 +(2 \log 2)k + 2k \log k + 2k \log \log k + \frac{5k \log \log k}{\log k}, \end{aligned}$$

then

4.1 $$\begin{aligned} \frac{1}{6}\cdot e^{\nu (k)/2} > \nu (k)^k,\qquad k\ge 7. \end{aligned}$$

Moreover, if $\mu > \nu (k)$ for some $k\ge 7$, then

4.2 $$\begin{aligned} \frac{1}{6}\cdot e^{\mu /2} > \mu ^k,\qquad k\ge 7. \end{aligned}$$

### Proof

Let $f(\mu ):=-\log 6+\mu /2-k\log \mu$. By $f'(\mu )=1/2-k/\mu$, *f* is increasing for $\mu >2k$. Hence the fact $\nu (k)>2k$ gives $f(\mu ) > f(\nu (k))$, and (4.2) follows from (4.1) which is equivalent to $f(\nu (k))>0$, $k\ge 7$. We set

$$\begin{aligned} {\overline{\nu }}(k):=-1+\frac{\nu (k)}{2 k \log k} = \frac{\log 6}{k \log k}+\frac{\log 2}{\log k} +\frac{\log \log k}{\log k}+\frac{5 \log \log k}{2 (\log k)^2}. \end{aligned}$$

The positivity of $f(\nu (k))$ is shown as follows:

$$\begin{aligned} f(\nu (k))&=-\log 6+\nu (k)/2 -k\log (2k \log k)-k\log (1+{\overline{\nu }}(k))\\&= \frac{5 k \log \log k}{2 \log k}-k\log (1+{\overline{\nu }}(k))\\&> k\Big ( \frac{5 \log \log k}{2 \log k}-{\overline{\nu }}(k) \Big )\qquad (\text {by } \log (1+x)<x \text { for }0<x)\\&= \frac{k}{2 \log k} \Big ( 3 \log \log k -\frac{2 \log 6}{k}-2 \log 2 -\frac{5 \log \log k}{\log k} \Big )\\&> \frac{k}{2 \log k} \Big (3 \log \log k -\frac{1}{5} -\frac{7}{5} -2 \Big ) = \frac{k}{2 \log k} \Big (3 \log \log k - \frac{18}{5} \Big ). \end{aligned}$$

The last inequality holds for all $k\ge 18$, because for such *k*

$$\begin{aligned} \frac{2 \log 6}{k}<\frac{1}{5}, \frac{5 \log \log k}{\log k}<2, \text { and } 2\log 2 <\frac{7}{5}. \end{aligned}$$

It is also straight-forward to prove $\log \log k >6/5$ for all $k\ge 28$. For the remaining cases $7\le k\le 27$ the inequality (4.1) is verified by numerical computation, which completes the proof of Lemma 4.1. $\square$

### Lemma 4.2

For $$k\ge 7$$ let

$$\begin{aligned} \kappa (k):=2\log 6+(2\log 2)k+ 2k \log k+2k\log \log k+\frac{2k\log \log k}{\log k}. \end{aligned}$$

Then we have

$$\begin{aligned} \frac{1}{6}e^{\kappa (k)/2}<\kappa (k)^k. \end{aligned}$$

### Proof

Let *f* defined as in Lemma 4.1, then the statement is equivalent to proving that

$$\begin{aligned} f(\kappa (k))=-\log 6+\frac{\kappa (k)}{2}-k\log \kappa (k)<0. \end{aligned}$$

Setting

$$\begin{aligned} {\tilde{\kappa }}(k):=-1+\frac{\kappa (k)}{2k\log k}=\frac{\log (6)}{k\log k}+\frac{\log 2}{\log k}+\frac{\log \log k}{\log k}+\frac{\log \log k}{(\log k)^2} \end{aligned}$$

we observe that

$$\begin{aligned} \begin{aligned} f(\kappa (k))&=-\log 6+\kappa (k)/2-k\log (2k\log k)-k\log (1+{\tilde{\kappa }}(k))\\&= \frac{2k \log \log k}{2\log k}-k\log (1+{\tilde{\kappa }}(k))\\&< \frac{k \log \log k}{\log k}-k({\tilde{\kappa }}(k)-{\tilde{\kappa }}(k)^2/2),\\ \end{aligned} \end{aligned}$$

because of $\log (1+x)>x-x^2/2$ for $x\in {\mathbb {R}}_{>0}$.

In order to show $f(\kappa (k))<0$, it would be enough therefore to show that $2\Bigl ({\tilde{\kappa }}(k)-\frac{\log \log k}{\log k}\Bigr )>{\tilde{\kappa }}^2$ below. We have

$$\begin{aligned} \begin{aligned}&2 \frac{\log 6 \log k +(\log 2)k \log k+k\log \log k}{k(\log k)^2}\\&\quad >\Bigl (\frac{\log 6\log k+(\log 2)k\log k+k(\log \log k)\log k+k\log \log k}{k(\log k)^2}\Bigr )^2, \end{aligned} \end{aligned}$$

which is equivalent to the inequality

$$\begin{aligned} \begin{aligned}&2 \log k\Bigl (\frac{\log 6}{k}+\log 2+\frac{\log \log k}{\log k}\Bigr )\\&\quad >(\log \log k)^2\Bigl (\frac{\log 6}{k\log \log k}+\frac{\log 2}{\log \log k}+1+\frac{1}{\log k}\Bigr )^2. \end{aligned} \end{aligned}$$

Since

$$\begin{aligned} 2 \log k\Bigl (\frac{\log 6}{k}+\log 2+\frac{\log \log k}{\log k}\Bigr )>(2\log 2)\log k>\frac{5}{4}\log k, \ k\ge 3, \end{aligned}$$

it suffices to show

$$\begin{aligned} \frac{5}{4}\log k>(\log \log k)^2\Bigl (\frac{\log 6}{k\log \log k}+\frac{\log 2}{\log \log k}+1+\frac{1}{\log k}\Bigr )^2, \end{aligned}$$

which after division by $(\log \log k)^2$ gives the equivalent inequality

$$\begin{aligned} \frac{5}{4}\frac{\log k}{(\log \log k)^2}>\Bigl (\frac{\log 6}{k\log \log k}+\frac{\log 2}{\log \log k}+1+\frac{1}{\log k}\Bigr )^2. \end{aligned}$$

Now note that $\frac{\log k}{(\log \log k)^2}$ is increasing and the right-hand side of the above inequality is decreasing for $k\ge \lceil e^{e^2}\rceil =1619$. Evaluating both sides at $k=e^{e^2}$ gives $\frac{5}{4}\frac{e^2}{4}>\frac{23}{10}$ for the left, and $\Bigl (1+\frac{1}{e^2}+\frac{\log 2}{2}+\frac{\log 6}{2e^{e^2}}\Bigr )^2<\frac{22}{10}$ for the right side. This proves the inequality for $k\ge 1619$. For $7\le k\le 1618$ the result follows by numerical evaluation. $\square$

### Definition 4.3

For $$k\ge 2$$ define

$$\begin{aligned} g(k):=\frac{3}{2\pi ^2}(\nu (k)^2+1), \end{aligned}$$

where $\nu (k)$ is as in Lemma 4.1.

### Theorem 4.4

For all $k\ge 2$ and $n> g(k)$ such that $(n,k)\ne (6,2)$ we have

4.3 $$\begin{aligned}&\frac{\sqrt{12}e^{\mu (n)}}{24n-1}\Bigl (1-\frac{1}{\mu (n)}-\frac{1}{\mu (n)^k}\Bigr )<p(n)\nonumber \\&\quad <\frac{\sqrt{12}e^{\mu (n)}}{24n-1}\Bigl (1-\frac{1}{\mu (n)}+\frac{1}{\mu (n)^k}\Bigr ). \end{aligned}$$

### Proof

From [1, p. 8, (2.9)] we find that

$$\begin{aligned} p(n)=\frac{\sqrt{12}e^{\mu (n)}}{24n-1}\Bigl (1-\frac{1}{\mu (n)}+T(n)\Bigr ) \text { for } n\ge 1, \end{aligned}$$

where *T*(*n*) is defined in [1, (2.10)]. In [1, (2.22)] it is proven that

4.4 $$\begin{aligned} |T(n)|<6e^{-\frac{\mu (n)}{2}} \text { for } n>350. \end{aligned}$$

By Lemma 4.1 we have that $\mu (n)^k<\frac{1}{6}e^{\frac{\mu (n)}{2}}$ for $k\ge 7$ and $\mu (n)>\nu (k)$, which is equivalent to

4.5 $$\begin{aligned} 6e^{-\frac{\mu (n)}{2}}<\frac{1}{\mu (n)^k} \text { for } \mu (n)>\nu (k). \end{aligned}$$

Since $\mu (n)=\frac{\pi }{6}\sqrt{24n-1}$, it follows that $\mu (n)> \nu (k)$ if and only if $n>g(k)$. Furthermore for $k\ge 7$, we have $g(k)>350$, this means that (4.4) is satisfied for $n>g(k)$.

By (4.4) and (4.5) we obtain that $|T(n)|<\frac{1}{\mu (n)^k}$ for $n>g(k)$ which proves that statement for $k\ge 7$. To prove the statement for $k\in \{2,\dots ,6\}$ we use the statement for $k=7$ which says that for all $n\ge \lceil g(7) \rceil =581$ we have

$$\begin{aligned} \frac{\sqrt{12}e^{\mu (n)}}{24n-1}\Bigl (1-\frac{1}{\mu (n)}-\frac{1}{\mu (n)^7}\Bigr )<p(n)<\frac{\sqrt{12}e^{\mu (n)}}{24n-1}\Bigl (1-\frac{1}{\mu (n)}+\frac{1}{\mu (n)^7}\Bigr ). \end{aligned}$$

However,

4.6 $$\begin{aligned} p(n)<\frac{\sqrt{12}e^{\mu (n)}}{24n-1}\Bigl (1-\frac{1}{\mu (n)}+\frac{1}{\mu (n)^7}\Bigr )<\frac{\sqrt{12}e^{\mu (n)}}{24n-1}\Bigl (1-\frac{1}{\mu (n)}+\frac{1}{\mu (n)^k}\Bigr )\qquad \end{aligned}$$

for $k\in \{2,\dots ,6\}$ and $n\ge 581$. To prove (4.6) for $g(k)<n<581$ it is enough to do a numerical evaluation of (4.6) for these values of *n* with the exception $n=6$ when $k=2$. We did this using computer algebra. Analogously, we see that for $k\in \{2,\dots ,6\}$ and $n\ge 581$ we have

4.7 $$\begin{aligned} \frac{\sqrt{12}e^{\mu (n)}}{24n-1}\Bigl (1-\frac{1}{\mu (n)}-\frac{1}{\mu (n)^k}\Bigr )<\frac{\sqrt{12}e^{\mu (n)}}{24n-1}\Bigl (1-\frac{1}{\mu (n)}-\frac{1}{\mu (n)^7}\Bigr )<p(n).\nonumber \\ \end{aligned}$$

In the same way we prove (4.7) for $g(k)<n<581$. $\square$

## Preparing for the proof of Theorem 6.6

In this section we prepare for the proof of our main theorem, Theorem 6.6, which is presented in Sect. 6. To this end, we need to introduce a variety of lemmas.

### Definition 5.1

For $y\in {\mathbb {R}}$, $0<y^2<24$, we define

$$\begin{aligned} G(y):=-\log \left( 1-\frac{y^2}{24}\right) +\frac{\pi }{6y}\sqrt{24}\left( \sqrt{1-\frac{y^2}{24}}-1\right) +\log \left( 1-\frac{y}{\frac{\pi }{6}\sqrt{24-y^2}}\right) , \end{aligned}$$

and its sequence of Taylor coefficients by

$$\begin{aligned} \sum _{u=1}^{\infty }g_uy^u:=G(y). \end{aligned}$$

### Definition 5.2

For $0<y^2<24$ and $i\in \{-1,1\}$, define

$$\begin{aligned} G_{i,k}(y):=G(y)+\log \left( 1+\frac{i\left( \frac{y}{\frac{\pi }{6}\sqrt{24-y^2}}\right) ^{k}}{1-\frac{y}{\frac{\pi }{6}\sqrt{24-y^2}}}\right) . \end{aligned}$$

### Lemma 5.3

Let *g*(*k*) be as in Definition 4.3. Then for all $k\ge 2$ and $n>g(k)$ with $(k,n)\ne (2,6)$ we have

$$\begin{aligned}&-\log 4\sqrt{3}-\log n +\pi \sqrt{\frac{2n}{3}}+G_{-1,k}(1/\sqrt{n})<\log p(n)\\&\quad < -\log 4\sqrt{3}-\log n +\pi \sqrt{\frac{2n}{3}}+G_{1,k}(1/\sqrt{n}). \end{aligned}$$

### Proof

Taking log of both sides of (4.3) gives

$$\begin{aligned} \log E_{-1,k}(n)<\log p(n)<\log E_{1,k}(n) \end{aligned}$$

where

$$\begin{aligned} E_{i,k}(n):=\log \sqrt{12} -\log (24n-1)+\mu (n)+ \log \Biggl (1-\frac{1}{\mu (n)}+\frac{i}{\mu (n)^{k}}\Biggr ). \end{aligned}$$

Now

$$\begin{aligned} \begin{aligned} E_{i,k}(n)=&\log \frac{\sqrt{12}}{24}-\log n-\log \left( 1-\frac{1}{24n}\right) +\pi \sqrt{\frac{2n}{3}}+\mu (n)\\&-\frac{\pi }{6}\sqrt{24n}+ \log \left( 1-\frac{1}{\mu (n)}+\frac{i}{\mu (n)^{k}}\right) \\ =&-\log 4\sqrt{3}-\log n +\pi \sqrt{\frac{2n}{3}}+R_{i,k}(n), \end{aligned} \end{aligned}$$

where

$$\begin{aligned} R_{i,k}(x):=-\log \left( 1-\frac{1}{24x}\right) +\mu (x)-\frac{\pi }{6}\sqrt{24x}+\log \left( 1-\frac{1}{\mu (x)}+\frac{i}{\mu (x)^{k}}\right) . \end{aligned}$$

Finally one verifies that $R_{i,k}(x)=G_{i,k}(1/\sqrt{x})$. $\square$

The quantity

$$\begin{aligned} \alpha :=\frac{\pi ^2}{36+\pi ^2} \end{aligned}$$

will play an important role in this and the next section.

### Lemma 5.4

Let $G(y)=\sum _{u=1}^{\infty }g_uy^u$ be the Taylor series expansion of *G*(*y*) as in Definition 5.1. Then

5.1 $$\begin{aligned} g_{2n}=\frac{1}{3^n2^{3n}n}-\frac{1}{2^{3n+1}3^nn}\left( -1+\frac{1}{\alpha ^n}\right) ,\ n\ge 1, \end{aligned}$$

and for $$n\ge 0$$,

5.2 $$\begin{aligned}&g_{2n+1}=\sqrt{6}\left[ (-1)^{n+1}\left( {\begin{array}{c}1/2\\ n+1\end{array}}\right) \frac{\pi }{2^{3n+3}3^{n+2}}\right. \nonumber \\&\qquad \qquad \quad \left. -\frac{1}{2^{3n+1}3^n\alpha ^n(1+2 n) \pi } \sum _{j=0}^n \alpha ^j\left( {\begin{array}{c}-\frac{1}{2}+j\\ j\end{array}}\right) \right] . \end{aligned}$$

### Proof

By using

$$\begin{aligned} \log \left( 1-\frac{y}{\frac{\pi }{6}\sqrt{24-y^2}}\right) =-\sum _{k=1}^{\infty }y^kk^{-1}\pi ^{-k}{6^k}24^{-k/2}\left( 1- \left( \frac{y}{\sqrt{24}}\right) ^2\right) ^{-k/2}, \end{aligned}$$

together with

$$\begin{aligned} \left( 1- \left( \frac{y}{\sqrt{24}}\right) ^2\right) ^{-k/2}=\sum _{n=0}^{\infty }(-1)^n\left( {\begin{array}{c}-k/2\\ n\end{array}}\right) \left( \frac{y}{\sqrt{24}}\right) ^{2n}, \end{aligned}$$

we obtain

$$\begin{aligned} g_{2n}=\frac{1}{3^n2^{3n}n}-\sum _{u=0}^{n-1}\frac{1}{3^{2u-n} 2^{n+2u}\pi ^{2n-2u}(2n-2u)}(-1)^u\left( {\begin{array}{c}u-n\\ u\end{array}}\right) , \ n\ge 1. \end{aligned}$$

For $$n\ge 0$$,

$$\begin{aligned} \begin{aligned} g_{2n+1}=&\sqrt{6}\Bigl [(-1)^{n+1}\left( {\begin{array}{c}1/2\\ n+1\end{array}}\right) \frac{\pi }{2^{3n+3}3^{n+2}}\\&-\sum _{u=0}^{n}\frac{1}{3^{2u-n} 2^{n+1+2u}\pi ^{2n+1-2u}(2n+1-2u)}(-1)^u\left( {\begin{array}{c}u-n-1/2\\ u\end{array}}\right) \Bigr ]. \end{aligned} \end{aligned}$$

Inputting this into the package Sigma developed by Carsten Schneider [13], we obtain (5.1) and (5.2). $\square$

We need various additional facts about the Taylor coefficients $g_u$ of *G*(*y*).

### Lemma 5.5

For $$0\le a<1$$,

$$\begin{aligned} \frac{a}{2}\le \sum _{j=1}^na^j\left( {\begin{array}{c}j-1/2\\ j\end{array}}\right) \le \frac{a}{2(1-a)}. \end{aligned}$$

### Proof

First we note that $$\left( {\begin{array}{c}j-1/2\\ j\end{array}}\right) =(-1)^j\left( {\begin{array}{c}-\frac{1}{2}\\ j\end{array}}\right) >0$$. Hence

$$\begin{aligned} \begin{aligned} \sum _{j=1}^{n}a^j\left( {\begin{array}{c}j-\frac{1}{2}\\ j\end{array}}\right) =&\sum _{j=1}^n(-a)^j\left( {\begin{array}{c}-\frac{1}{2}\\ j\end{array}}\right) =\sum _{j=0}^n(-a)^j\left( {\begin{array}{c}-\frac{1}{2}\\ j\end{array}}\right) -1\\ <&\sum _{j=0}^{\infty }(-a)^j\left( {\begin{array}{c}-\frac{1}{2}\\ j\end{array}}\right) -1=\frac{1}{\sqrt{1-a}}-1\le \frac{a}{2(1-a)}. \end{aligned} \end{aligned}$$

This proves the upper bound. To prove the lower bound note that the first term of the sum is $\frac{a}{2}$ and the other terms are all positive. $\square$

### Lemma 5.6

Let $$s_n:=(-1)^n\left( {\begin{array}{c}1/2\\ n+1\end{array}}\right) $$. For $n\ge 0$ we have $s_n\ge 0$ and $s_n$ is a decreasing sequence, that is $s_n>s_{n+1}$ for all $n\ge 0$.

### Lemma 5.7

For $n\ge 0$ we have

$$\begin{aligned}&-\frac{\sqrt{6}}{2\pi 2^{3n}3^{n}\alpha ^n(1+2n)}\left( 1+\frac{\alpha }{2}\right) \\&\ge g_{2n+1}\ge -\frac{\sqrt{6}}{2\pi 2^{3n}3^n\alpha ^n(1+2n)}\left( \frac{\pi ^2}{72}+1+\frac{\alpha }{2(1-\alpha )}\right) . \end{aligned}$$

### Proof

From Lemmas 5.4, 5.5, and 5.6, we obtain

$$\begin{aligned} -\frac{\sqrt{6}}{2\pi 2^{3n}3^{n}\alpha ^n(1+2n)}\left( 1+\frac{\alpha }{2}\right) \ge g_{2n+1}. \end{aligned}$$

Again by Lemmas 5.4, 5.5, and 5.6, we have

$$\begin{aligned} \begin{aligned} g_{2n+1}\ge&-\frac{\sqrt{6}}{2^{3n}3^n}\Bigr (\frac{\pi }{72}(-1)^{0+1}\left( {\begin{array}{c}1/2\\ 0+1\end{array}}\right) +\frac{1+\frac{\alpha }{2(1-\alpha )}}{2\pi \alpha ^n(1+2n)}\Bigr )\\ =&-\frac{\sqrt{6}}{2\pi 2^{3n}3^n\alpha ^n(1+2n)}\Bigl (\frac{\pi ^2\alpha ^n(1+2n)}{72}+1+\frac{\alpha }{2(1-\alpha )}\Bigr )\\ \ge&-\frac{\sqrt{6}}{2\pi 2^{3n}3^n\alpha ^n(1+2n)}\Bigl (\frac{\pi ^2\alpha ^0(1+2\cdot 0)}{72}+1+\frac{\alpha }{2(1-\alpha )}\Bigr ). \end{aligned} \end{aligned}$$

The last line is because $\alpha ^n(1+2n)$ is a decreasing sequence of *n* for $n\ge 0$. $\square$

### Lemma 5.8

For $n\ge 1$ we have

$$\begin{aligned} -\frac{1}{3^n2^{3n+1}n \alpha ^n}\le g_{2n}\le \frac{1}{3^n2^{3n}n \alpha ^n}\Bigl (\frac{3\alpha }{2}-\frac{1}{2}\Bigr ). \end{aligned}$$

### Proof

By Lemma 5.4 the statement follows from

$$\begin{aligned} \begin{aligned} g_{2n}=&\frac{1}{3^n2^{3n}n}-\frac{1}{2^{3n+1}3^nn}\Bigl (-1+\frac{1}{\alpha ^n}\Bigr )=\frac{1}{3^n2^{3n}\alpha ^nn}\Bigl (\frac{3\alpha ^n}{2}-\frac{1}{2}\Bigr ). \end{aligned} \end{aligned}$$

$$\square $$

### Lemma 5.9

Define

$$\begin{aligned} \mu _1:=\frac{\sqrt{6}}{2\pi }\Bigl (\frac{\pi ^2}{72}+1+\frac{\alpha }{2(1-\alpha )}\Bigr ) \ \ \text { and } \ \ \mu _2:=\frac{\sqrt{6}}{2\pi }\Bigl (1+\frac{\alpha }{2}\Bigr ). \end{aligned}$$

Then for $m\ge 0$ and $0<y\le \epsilon < 2\sqrt{6\alpha }$,

$$\begin{aligned}&-\frac{\mu _2}{2^{3m}3^m\alpha ^m(1+2m)}y^{2m+1}\\&\ge \sum _{n=m}^{\infty }g_{2n+1}y^{2n+1}\\&\ge -\frac{\mu _1 }{2^{3m}3^{m}\alpha ^m(1+2m)}\frac{1}{1-\frac{\epsilon ^2}{3\alpha \cdot 2^3}}y^{2m+1}. \end{aligned}$$

### Proof

By Lemma 5.7 we have

$$\begin{aligned} \begin{aligned} \sum _{n=m}^{\infty }g_{2n+1}y^{2n+1}&\ge -\mu _1\sum _{n=m}^{\infty }\frac{1}{2^{3n}3^n\alpha ^n(1+2n)}y^{2n+1}\\&\ge -\frac{\mu _1 y^{2m+1}}{1+2m}\sum _{n=0}^{\infty } \frac{1}{2^{3(n+m)}3^{n+m}\alpha ^{n+m}}y^{2n}\\&=-\frac{\mu _1 y^{2m+1}}{2^{3m}3^{m}\alpha ^m(1+2m)}\frac{1}{1-\frac{y^2}{3\alpha \cdot 2^3}}\\&\ge -\frac{\mu _1 }{2^{3m}3^{m}\alpha ^m(1+2m)}\frac{1}{1-\frac{\epsilon ^2}{3\alpha \cdot 2^3}}y^{2m+1},\\ \end{aligned} \end{aligned}$$

and again by Lemma 5.7 we have

$$\begin{aligned} \begin{aligned}&\sum _{n=m}^{\infty }g_{2n+1}y^{2n+1}\le -\mu _2\sum _{n=m}^{\infty }\frac{y^{2n+1}}{2^{3n}3^n\alpha ^n(1+2n)}\le -\mu _2\frac{y^{2m+1}}{2^{3m}3^m\alpha ^m(1+2m)}. \end{aligned} \end{aligned}$$

$$\square $$

### Lemma 5.10

For $$m\ge 1$$ and $$0<y\le \epsilon <2\sqrt{6\alpha }$$,

$$\begin{aligned} \frac{3\alpha -1}{3^m2^{3m+1}m\alpha ^m}y^{2m} \ge \sum _{n=m}^{\infty }g_{2n}y^{2n}\ge -y^{2m}\frac{1}{3^m2^{3m+1}m\alpha ^m}\frac{1}{1-\frac{\epsilon ^2}{3\cdot 2^3\cdot \alpha }}. \end{aligned}$$

### Proof

By Lemma 5.8,

$$\begin{aligned} \begin{aligned} \sum _{n=m}^{\infty }g_{2n}y^{2n}\ge&-\frac{1}{2}\sum _{n=m}^{\infty }\frac{1}{3^n2^{3n}n \alpha ^n}y^{2n}\ge -y^{2m}\frac{1}{2}\sum _{n=m}^{\infty }\frac{1}{3^n2^{3n}m \alpha ^n}y^{2n-2m}\\ =&-y^{2m}\frac{1}{3^m2^{3m+1}m\alpha ^m}\frac{1}{1-\frac{y^2}{3\cdot 2^3\cdot \alpha }}\ge -y^{2m}\frac{1}{3^m2^{3m+1}m\alpha ^m}\frac{1}{1-\frac{\epsilon ^2}{3\cdot 2^3\cdot \alpha }}. \end{aligned} \end{aligned}$$

Again by Lemma 5.8,

$$\begin{aligned} \begin{aligned}&\sum _{n=m}^{\infty }g_{2n}y^{2n}\le \frac{3\alpha -1}{2} \sum _{n=m}^{\infty }\frac{1}{3^n2^{3n}n \alpha ^n}y^{2n}\le \frac{3\alpha -1}{2} \frac{1}{3^m2^{3m}m \alpha ^m}y^{2m}. \end{aligned} \end{aligned}$$

$$\square $$

### Definition 5.11

For $$0<y\le \epsilon <1$$ define

5.3 $$\begin{aligned} B(y):=\frac{y}{\frac{\pi }{6}\sqrt{24-y^2}} \quad \text { and } \quad B_{\epsilon ,k}:=\epsilon ^{-k}\frac{B(\epsilon )^k}{1-B(\epsilon )}. \end{aligned}$$

### Lemma 5.12

If $$0<y\le \epsilon <1$$, then

$$\begin{aligned} \log \Bigl (1+\frac{B(y)^{k}}{1-B(y)}\Bigr )\le \frac{B_{\epsilon ,k}}{1-(B_{\epsilon ,k}\epsilon ^{k})^2}y^{k}, \ k\ge 1. \end{aligned}$$

### Proof

First note that for $0<y<\sqrt{24}$ the function *B*(*y*) is increasing and also that $\frac{B(y)^{k}}{1-B(y)}\le \frac{B(y)^{k}}{1-B(\epsilon )}$ and $B(y)<\frac{y}{\frac{\pi }{6}\sqrt{24-\epsilon ^2}}=\epsilon ^{-1}yB(\epsilon )$. Hence

$$\begin{aligned} \frac{B(y)^{k}}{1-B(\epsilon )}<\frac{\epsilon ^{-k}y^kB(\epsilon )^k}{1-B(\epsilon )}=B_{\epsilon ,k}y^{k}. \end{aligned}$$

Consequently,

$$\begin{aligned} \begin{aligned} \log \left( 1+\frac{B(y)^{k}}{1-B(y)}\right)&\le \log \left( 1+B_{\epsilon ,k}y^{k}\right) =-\sum _{n=1}^{\infty }\frac{(-1)^n}{n}B_{\epsilon ,k}^ny^{kn}\\&=-\sum _{n=1}^{\infty }\frac{1}{2n}B_{\epsilon ,k}^{2n}y^{2kn}+\sum _{n=0}^{\infty }\frac{1}{2n+1}B_{\epsilon ,k}^{2n+1}y^{k(2n+1)}\\&\le \sum _{n=0}^{\infty }\frac{1}{2n+1}B_{\epsilon ,k}^{2n+1}y^{k(2n+1)}\le \sum _{n=0}^{\infty }B_{\epsilon ,k}^{2n+1}y^{k(2n+1)}\\&=\frac{B_{\epsilon ,k}y^{k}}{1-(B_{\epsilon ,k}y^{k})^2}\le \frac{B_{\epsilon ,k}}{1-(B_{\epsilon ,k}\epsilon ^{k})^2}y^{k}. \end{aligned} \end{aligned}$$

$$\square $$

### Lemma 5.13

If $$0<y\le \epsilon <1$$, then

$$\begin{aligned} \log \Bigl (1-\frac{B(y)^{k}}{1-B(y)}\Bigr )\ge -\frac{B_{\epsilon ,k}}{1-B_{\epsilon ,k}\epsilon ^{k}}y^{k}, \ k\ge 1. \end{aligned}$$

### Proof

$$\begin{aligned} \begin{aligned} \log \Bigl (1-\frac{B(y)^{k}}{1-B(y)}\Bigr )\ge&\log \Bigl (1-B_{\epsilon ,k}y^{k})=-\sum _{n=1}^{\infty }\frac{1}{n}B_{\epsilon ,k}^ny^{kn}\ge -\sum _{n=1}^{\infty }B_{\epsilon ,k}^ny^{kn} \\ =&-\frac{B_{\epsilon ,k}y^{k}}{1-B_{\epsilon ,k}y^{k}}\ge -\frac{B_{\epsilon ,k}}{1-B_{\epsilon ,k}\epsilon ^{k}}y^{k}. \end{aligned} \end{aligned}$$

$$\square $$

### Lemma 5.14

For all $k\ge 2$ and $0<\epsilon \le \frac{1}{\sqrt{7}}$ we have

$$\begin{aligned} \frac{6^k}{5^k\pi ^k}<B_{\epsilon ,k}\le \frac{b_0\cdot 6^k}{\pi ^k(\sqrt{24-\frac{1}{7}})^k}, \end{aligned}$$

where $b_0:=\frac{1}{1-\frac{6}{\sqrt{7}\pi \sqrt{24-\frac{1}{7}}}}$ and again $B_{\epsilon ,k}$ as in (5.3).

### Proof

Define

$$\begin{aligned} s:=\sqrt{24-\epsilon ^2}, \ l_s:=\sqrt{24-\frac{1}{7}}, \ u_s:=4.9, \ l_{\epsilon }:= 0, \text { and } u_{\epsilon }:=\frac{1}{\sqrt{7}}. \end{aligned}$$

For all $k\ge 2$ and $0<\epsilon \le \frac{1}{\sqrt{7}}$, we have

$$\begin{aligned} l_s\le s< u_s \quad \text { and }\quad l_{\epsilon }<\epsilon \le u_{\epsilon }. \end{aligned}$$

The following conventions for the letters *l* and *u* will be useful: $l_a$ denotes a lower bound for the quantity *a*, and $u_a$ will denote an upper bound for the quantity *a*. And again we use *B*(*y*) as defined in Definition 5.11.

Then

$$\begin{aligned} 0=\frac{l_{\epsilon }}{\frac{\pi }{6}{u_s}}<B(\epsilon )=\frac{\epsilon }{\frac{\pi }{6}s}\le \frac{u_{\epsilon }}{\frac{\pi }{6}l_s}. \end{aligned}$$

Let us define $l_B:=0$ and $u_B:= \frac{u_{\epsilon }}{\frac{\pi }{6}l_s}$. Then

$$\begin{aligned} \begin{aligned} l_B<B(\epsilon )\le u_B&\Rightarrow 1-u_B\le 1-B(\epsilon )<1-l_B=1\\&\Rightarrow \frac{1}{1-l_B}=1<\frac{1}{1-B(\epsilon )}\le \frac{1}{1-u_B}, \end{aligned} \end{aligned}$$

and $$\frac{1}{(\frac{\pi }{6}u_s)^k}<\frac{1}{(\frac{\pi }{6}s)^k}\le \frac{1}{(\frac{\pi }{6}l_s)^k}$$. Hence

$$\begin{aligned} \begin{aligned} \frac{6^k}{5^k\pi ^k}&<\frac{6^k}{(4.9)^k\pi ^k}=\frac{1}{(1-l_B)(\frac{\pi }{6}u_s)^k}<B_{\epsilon ,k}\le \frac{1}{(1-u_B)(\frac{\pi }{6}l_s)^k}\\&=\frac{1}{\left( 1-\frac{\frac{1}{\sqrt{7}}}{\frac{\pi }{6}\sqrt{24-\frac{1}{7}}}\right) \left( \frac{\pi ^k}{6^k}\left( \sqrt{24-\frac{1}{7}}\right) ^k\right) }=\frac{b_0}{\frac{\pi ^k}{6^k}\left( \sqrt{24-\frac{1}{7}}\right) ^k}. \end{aligned} \end{aligned}$$

$$\square $$

### Definition 5.15

Define

$$\begin{aligned} \beta :=\sqrt{24-\frac{1}{7}} \end{aligned}$$

and for $$k\ge 0$$,

$$\begin{aligned} C_k:=\frac{6^k}{(\pi \beta )^k}. \end{aligned}$$

### Lemma 5.16

Let $0<\epsilon \le \frac{1}{\sqrt{7}}$ and $B_{\epsilon ,k}$ be as in (5.3). Then for $k\ge 2$,

$$\begin{aligned} \frac{B_{\epsilon ,k}}{1-(B_{\epsilon ,k}\epsilon ^k)^2}\le b_1B_{\epsilon ,k} \quad \text { and }\quad \frac{B_{\epsilon ,k}}{1-B_{\epsilon ,k}\epsilon ^k}\le b_2B_{\epsilon ,k}, \end{aligned}$$

with

$$\begin{aligned} b_1:=\frac{1}{1-\frac{1}{49}b_0^2C_4} \quad \text {, }b_2:=\frac{1}{1-\frac{1}{7}b_0C_2}, \end{aligned}$$

and $b_0$ as in Lemma 5.14.

### Proof

We obtain, using Lemma 5.14,

$$\begin{aligned} \frac{B_{\epsilon ,k}}{1-B_{\epsilon ,k}\epsilon ^k}\le \frac{B_{\epsilon ,k}}{1-\frac{1}{7}B_{\epsilon ,k}}\le \frac{B_{\epsilon ,k}}{1-\frac{1}{7}b_0C_2}=b_2B_{\epsilon ,k}, \end{aligned}$$

and

$$\begin{aligned} \frac{B_{\epsilon ,k}}{1-(B_{\epsilon ,k}\epsilon ^k)^2}\le \frac{B_{\epsilon ,k}}{1-\frac{1}{49}B_{\epsilon ,k}^2}\le \frac{B_{\epsilon ,k}}{1-\frac{1}{49}b_0^2C_4}=b_1B_{\epsilon ,k}. \end{aligned}$$

$$\square $$

### Lemma 5.17

Let $C_k$ be as in Definition 5.15, then

$$\begin{aligned} C_{2m}<\frac{1}{3^m 2^{3m}\alpha ^m m}, \ m\ge 10, \text { and } C_{2m-1}<\frac{69}{25}\frac{1}{ 2^{3m}3^m\alpha ^m(2m-1)}, \ m\ge 14. \end{aligned}$$

### Proof

We start with the first inequality:

$$\begin{aligned} \begin{aligned}&C_{2m}=\Bigl (\frac{252}{167\pi ^2}\Bigr )^m<\frac{(36+\pi ^2)^m}{3^m2^{3m}m\pi ^{2m}}\Leftrightarrow \Bigl (\frac{6048}{6012+167\pi ^2}\Bigr )^m m<1. \end{aligned} \end{aligned}$$

To prove the inequality in the rewritten form, define $\ell :=\frac{6048}{6012+167\pi ^2}$ and note that $\ell <1$. Moreover, for $m\ge 10$,

$$\begin{aligned} m\ell ^m<1 \Leftrightarrow \log m+m\log \ell <0. \end{aligned}$$

Define $f(m):=m\log \ell +\log m$. We have to show $f(m)<0$ for all $m\ge 10$. We first show that *f*(*m*) is decreasing for $m\ge 10$. This is equivalent to $f'(m)=\log \ell +\frac{1}{m}<0$ for $m\ge 10$. This is equivalent to showing $\ell e^{1/m}<1$ for $m\ge 10$.

Now for $m\ge 10$ we have $\ell e^{1/m}\le \ell e^{1/10}.$

By numerics, $\ell e^{1/10}<1$ and $f(10)<0$. Since *f*(*m*) is decreasing and $f(m)\le f(10)<0$ for $m\ge 10$, the first inequality is proven. Now for the second inequality, first note that

$$\begin{aligned} C_{2m-1}=\Bigl (\frac{6}{\pi \beta }\Bigr )^{2m-1}=\Bigl (\frac{252}{167\pi ^2}\Bigr )^m\Bigl (\frac{\pi }{6}\sqrt{\frac{167}{7}}\Bigr ). \end{aligned}$$

Hence we have to show

$$\begin{aligned} \begin{aligned}&\Bigl (\frac{252}{167\pi ^2}\Bigr )^m\Bigl (\frac{\pi }{6}\sqrt{\frac{167}{7}}\Bigr )<\frac{69}{25}\frac{1}{2^{3m}3^m\alpha ^m(2m-1)}, \end{aligned} \end{aligned}$$

which is equivalent to

$$\begin{aligned} \begin{aligned}&\Bigl (\frac{6048}{6012+167\pi ^2}\Bigr )^m(2m-1)<\frac{414}{25\pi }\sqrt{\frac{7}{167}}\Leftrightarrow (2m-1)\ell ^m<\frac{414}{25\pi }\sqrt{\frac{7}{167}}\\&\Leftrightarrow \underbrace{m\log \ell +\log (2m-1)-\log \Bigl (\frac{414}{25\pi }\sqrt{\frac{7}{167}}\Bigr )}_{=:g(m)}<0. \end{aligned} \end{aligned}$$

Now analogously to the proof of the first case one observes that *g*(*m*) is decreasing for $m\ge 14$ and that $g(14)<0$, hence $g(m)\le g(14)<0$. $\square$

## Proofs of Theorems 6.6 and 1.1

After the preparations made in Sect. 5, in this section we prove our Main Theorem, Theorem 6.6, which implies Theorem 1.1 as a corollary. Again we let

$$\begin{aligned} \alpha =\frac{\pi ^2}{36+\pi ^2}. \end{aligned}$$

### Definition 6.1

Let $B_{\epsilon ,k}$ be as in Definition 5.11 and $\mu _1,\mu _2$ as in Lemma 5.9 and $\nu :=\frac{3\alpha -1}{2}$. Moreover, let $0<\epsilon \le \frac{1}{\sqrt{7}}$. For $m,k\ge 1$ we define

$$\begin{aligned} A_{1,k}(2m):= & {} \frac{B_{\epsilon ,k}}{1-(B_{\epsilon ,k}\epsilon ^{k})^2}\epsilon ^{k-2m}+\nu \frac{1}{3^m2^{3m}m \alpha ^m},\\ A_{-1,k}(2m):= & {} \frac{B_{\epsilon ,k}}{1-B_{\epsilon ,k}\epsilon ^{k}} \epsilon ^{k-2m}+\frac{1}{3^m2^{3m+1}m\alpha ^m}\frac{1}{1-\frac{\epsilon ^2}{3\cdot 2^3\alpha }}\\&+\frac{\mu _1}{2^{3m}3^m\alpha ^m(1+2m)}\frac{1}{1-\frac{\epsilon ^2}{3\alpha \cdot 2^3}},\\ A_{1,k}(2m-1):= & {} \frac{B_{\epsilon ,k}}{1-(B_{\epsilon ,k}\epsilon ^{k})^2}\epsilon ^{k-2m+1}-\frac{\mu _2}{2^{3m-3}3^{m-1}\alpha ^{m-1}(2m-1)},\\ A_{-1,k}(2m-1):= & {} \frac{B_{\epsilon ,k}}{1-B_{\epsilon ,k}\epsilon ^{k}}\epsilon ^{k-2m+1}+\frac{1}{3^m2^{3m+1}m\alpha ^m}\frac{1}{1-\frac{\epsilon ^2}{3\cdot 2^3\alpha }},\\&+\frac{\mu _1}{2^{3m-3}3^{m-1}\alpha ^{m-1}(2m-1)}\frac{1}{1-\frac{\epsilon ^2}{3\alpha \cdot 2^3}}. \end{aligned}$$

### Lemma 6.2

Let $\sum _{n=1}^{\infty }g_ny^n$ as in Definition 5.1 and $G_{i,k}(y)$ as in Definition 5.2. Moreover let $0<y\le \epsilon \le \frac{1}{\sqrt{7}}$. Then for $k\ge 2m\ge 2$, we have

$$\begin{aligned} \sum _{n=1}^{2m-1}g_ny^n-A_{-1,k}(2m)y^{2m}\le & {} G_{-1,k}(y) \text { and } G_{1,k}(y) \le \sum _{n=1}^{2m-1}g_ny^n+A_{1,k}(2m)y^{2m}, \end{aligned}$$

and for $$k\ge 2m-1\ge 1$$,

$$\begin{aligned}&\sum _{n=1}^{2m-2}g_ny^n-A_{-1,k}(2m-1)y^{2m-1} \le G_{-1,k}(y) \text { and }\\&G_{1,k}(y)\le \sum _{n=1}^{2m-2}g_ny^n +A_{1,k}(2m-1)y^{2m-1}. \end{aligned}$$

### Proof

For $k\ge 2m\ge 2$, by using the Lemmas 5.9 to 5.12, we obtain

$$\begin{aligned} \begin{aligned} G_{1,k}(y)&\le \sum _{n=1}^{2m-1}g_ny^n+\frac{B_{\epsilon ,k}}{1-(B_{\epsilon ,k}\epsilon ^{k})^2}y^{k}+\nu \frac{1}{3^m2^{3m}m \alpha ^m}y^{2m}\\&\quad -\frac{\mu _2}{2^{3m}3^m\alpha ^m(1+2m)}y^{2m+1}\\&\le \sum _{n=1}^{2m-1}g_ny^n+\frac{B_{\epsilon ,k}}{1-(B_{\epsilon ,k}\epsilon ^{k})^2}\epsilon ^{k-2m}y^{2m}+\nu \frac{1}{3^m2^{3m}m \alpha ^m}y^{2m}\\&=\sum _{n=1}^{2m-1}g_ny^n+A_{1,k}(2m)y^{2m}.\\ \end{aligned} \end{aligned}$$

By using the Lemmas 5.9 to 5.10 together with Lemma 5.13 we obtain

$$\begin{aligned} G_{-1,k}(y)\ge & {} \sum _{n=1}^{2m-1}g_ny^n-\frac{B_{\epsilon ,k}}{1-B_{\epsilon ,k}\epsilon ^{k}}y^{k}-\frac{1}{3^m2^{3m+1}m\alpha ^m}\frac{1}{1-\frac{\epsilon ^2}{3\cdot 2^3\alpha }}y^{2m}\\&-\frac{\mu _1}{2^{3m}3^m\alpha ^m(1+2m)}\frac{1}{1-\frac{\epsilon ^2}{3\alpha \cdot 2^3}}y^{2m+1}\\\ge & {} \sum _{n=1}^{2m-1}g_ny^n-\frac{B_{\epsilon ,k}}{1-B_{\epsilon ,k}\epsilon ^{k}}\epsilon ^{k-2m}y^{2m}-\frac{1}{3^m2^{3m+1}m\alpha ^m}\frac{1}{1-\frac{\epsilon ^2}{3\cdot 2^3\alpha }}y^{2m}\\&-\frac{\mu _1}{2^{3m}3^m\alpha ^m(1+2m)}\frac{1}{1-\frac{\epsilon ^2}{3\alpha \cdot 2^3}}y^{2m}\\= & {} \sum _{n=1}^{2m-1}g_ny^n-A_{-1,k}(2m)y^{2m}. \end{aligned}$$

The statement for $A_{-1,k}(2m-1)$ is proven analogously. $\square$

### Lemma 6.3

We have for $m\ge 10$ that

$$\begin{aligned} A_{1,k}(2m)<\frac{1}{3^m2^{3m}m \alpha ^m}, \quad \ A_{-1,k}(2m)<\frac{2}{3^m2^{3m}m \alpha ^m} \end{aligned}$$

and for $$m\ge 14$$

$$\begin{aligned} A_{1,k}(2m-1)<\frac{2}{3^m2^{3m}(2m-1) \alpha ^m}, \quad \ A_{-1,k}(2m-1)<\frac{7}{3^m2^{3m}(2m-1) \alpha ^m}. \end{aligned}$$

### Proof

For $m\ge 10$ we have

$$\begin{aligned} A_{1,k}(2m)= & {} \ \frac{B_{\epsilon ,k}}{1-(B_{\epsilon ,k}\epsilon ^k)^2}\epsilon ^{k-2m}+\nu \frac{1}{3^m2^{3m}\alpha ^mm}\text { (by Definition } \mathrm{6.1}\mathrm{)}\\< & {} \ b_1B_{\epsilon ,k}\epsilon ^{k-2m}+\nu \frac{1}{3^m2^{3m}\alpha ^mm}\text { (by Lemma } \mathrm{5.16}\mathrm{)}\\< & {} \ b_1b_0\frac{6^k}{(\pi \beta )^k}\epsilon ^{k-2m}+\nu \frac{1}{3^m2^{3m}\alpha ^mm}\text { (by Lemma } \mathrm{5.14}\mathrm{)}\\= & {} \ b_0b_1C_k\epsilon ^{k-2m}+\nu \frac{1}{3^m2^{3m}\alpha ^mm}\text { (using Definition } \mathrm{5.15}\mathrm{)}\\\le & {} \ b_0b_1C_{2m}+\nu \frac{1}{3^m2^{3m}\alpha ^mm}\\&\text { (because } f(k):=C_k\epsilon ^{k-2m}\text { is decreasing for all } k\ge 2m)\\< & {} \ b_0b_1\frac{1}{3^m2^{3m}\alpha ^mm}+\nu \frac{1}{3^m2^{3m}\alpha ^mm}\text { (by Lemma }\mathrm{5.17}\mathrm{)}\\= & {} \ \Bigl (b_0b_1+\nu \Bigr )\frac{1}{3^m2^{3m}\alpha ^mm}\\< & {} \ \frac{1}{3^m2^{3m}\alpha ^mm} \text { (by evaluating } b_0b_1+\nu \text { numerically). } \end{aligned}$$

Similarly,

$$\begin{aligned}&A_{-1,k}(2m)\\&\quad = \ \frac{B_{\epsilon ,k}}{1-B_{\epsilon ,k}\epsilon ^k}\epsilon ^{k-2m} +\frac{1}{3^m2^{3m+1}\alpha ^m m}\frac{1}{1-\frac{\epsilon ^2}{24\alpha }}\\&\qquad +\frac{\mu _1}{2^{3m}3^m\alpha ^m(2m+1)}\frac{1}{1-\frac{\epsilon ^2}{24\alpha }} \text { (by Definition } \mathrm{6.1}\mathrm{)}\\&\quad< \ b_2B_{\epsilon ,k}\epsilon ^{k-2m}+\frac{1}{2}\frac{1}{3^m2^{3m}\alpha ^mm}\frac{1}{1-\frac{\epsilon ^2}{24\alpha }}+\frac{\mu _1}{2^{3m}3^m\alpha ^m(2m+1)}\frac{1}{1-\frac{\epsilon ^2}{24\alpha }}\\&\quad \text { (by Lemma } \mathrm{5.16}\mathrm{)}\\&\quad< \ b_2b_0\frac{6^k}{(\pi \beta )^k}\epsilon ^{k-2m}+\frac{1}{2}\frac{1}{3^m2^{3m}\alpha ^mm}\frac{1}{1-\frac{\epsilon ^2}{24\alpha }}+\frac{\mu _1}{2^{3m}3^m\alpha ^m(2m+1)}\frac{1}{1-\frac{\epsilon ^2}{24\alpha }}\\&\quad \text { (by Lemma } \mathrm{5.14}\mathrm{)}\\&\quad \le \ b_0b_2\cdot C_{2m}+\frac{1}{2}\frac{1}{3^m2^{3m}\alpha ^mm}\frac{1}{1-\frac{1}{168\alpha }}+\frac{\mu _1}{2^{3m}3^m\alpha ^m(2m+1)}\frac{1}{1-\frac{1}{168\alpha }}\\&\quad< \ b_0b_2\frac{1}{3^m2^{3m}\alpha ^mm}+\frac{1}{2}\frac{1}{3^m2^{3m}\alpha ^mm}\frac{1}{1-\frac{1}{168\alpha }}+\frac{1}{2}\frac{\mu _1}{2^{3m}3^m\alpha ^mm}\frac{1}{1-\frac{1}{168\alpha }}\\&\quad \text { (by Lemma } \mathrm{5.17}\mathrm{)}\\&\quad = \ \Bigl (b_0b_2+\frac{1}{2}\frac{1}{1-\frac{1}{168\alpha }}(1+\mu _1)\Bigr )\frac{1}{3^m2^{3m}\alpha ^mm}\\&\quad < \ \frac{2}{3^m2^{3m}\alpha ^mm} \text { (by evaluating } b_0b_2+\frac{1}{2}\frac{1}{1-\frac{1}{168\alpha }}(1+\mu _1) \text { numerically).} \end{aligned}$$

The statements for $A_{1,k}(2m-1)$ and $A_{-1,k}(2m-1)$ are proven analogously. $\square$

### Definition 6.4

For $n,U\ge 1$ we define

$$\begin{aligned} P_n(U):=-\log 4\sqrt{3}-\log n +\pi \sqrt{\frac{2n}{3}}+\sum _{u=1}^{U}g_u(1/\sqrt{n})^u. \end{aligned}$$

### Lemma 6.5

Let *g*(*k*) be as in Definition 4.3 and $P_n(U)$ as in Definition 6.4. If $m\ge 1$, $k\ge 2m$ and

$$\begin{aligned} n> \left\{ \begin{array}{cc} 6 &{} \text {if } m=1, \\ g(k) &{} \text {if } m\ge 2,\end{array}\right. \end{aligned}$$

then

6.1 $$\begin{aligned} -A_{-1,k}(2m)\frac{1}{n^m}<\log p(n)-P_n(2m-1)< A_{1,k}(2m)\frac{1}{n^{m}}. \end{aligned}$$

If $m\ge 2$, $k\ge 2m-1$, and $n>g(k)$, then

6.2 $$\begin{aligned} -A_{-1,k}(2m-1)\frac{1}{n^{m-\frac{1}{2}}}<\log p(n)-P_n(2m-2)<A_{1,k}(2m-1)\frac{1}{n^{m-\frac{1}{2}}}.\qquad \end{aligned}$$

### Proof

We start with the inequality from Lemma 5.3. Next we use Lemma 6.2 to bound $G_{1,k}(y)$. Finally we set $y=\frac{1}{\sqrt{n}}$ and obtain the desired result. $\square$

### Theorem 6.6

Let $G(y)=\sum _{n=1}^{\infty }g_ny^n$ be as in Definition 5.1. Let *g*(*k*) be as in Definition 4.3 and $P_n(U)$ as in Definition 6.4. If $m\ge 1$ and $n>g(2m)$, then

6.3 $$\begin{aligned} P_n(2m-1)-\frac{2}{3^m2^{3m}\alpha ^mmn^m}< \log p(n)< P_n(2m-1)+\frac{1}{3^m2^{3m}\alpha ^mmn^{m}};\nonumber \\ \end{aligned}$$

if $m\ge 2$ and $n>g(2m-1)$, then

6.4 $$\begin{aligned}&P_n(2m-2)-\frac{7}{3^m2^{3m}\alpha ^m(2m-1)n^{m-1/2}}<\log p(n)\nonumber \\&\quad < P_n(2m-2)+\frac{2}{3^m2^{3m}\alpha ^m(2m-1)n^{m-1/2}}. \end{aligned}$$

### Proof

We start by setting $k=2m$ in (6.1) of Lemma 6.5, and $k=2m-1$ in (6.2). In this inequality we bound $A_{1,k}(m)$ resp $A_{-1,k}(m)$ by using Lemma 6.3. This gives (6.4) for all $m\ge 14$ and $n>g(2m-1)$, and (6.3) for $m\ge 10$ and $n>g(2m)$.

In order to prove (6.3) and (6.4) for the remaining values of *m*, firstly we will prove that

6.5 $$\begin{aligned}&\text { if } \mathrm{(6.3)} \text { holds for } m\ge 2 \text { and all } n\ge y\ge 1,\nonumber \\&\text { then } \mathrm{(6.3)} \text { holds for } m-1 \text { and all } n\ge y. \end{aligned}$$

In particular, if we subtract from the lower bound on $\log p(n)$ with parameter *m* in (6.3) the lower bound on $\log p(n)$ with parameter $m-1$, we obtain $f(2m,-4)-g(2m-2,-4)$, where

$$\begin{aligned} f(w,x):=\sum _{u=w-2}^{w-1}g_u\Bigl (\frac{1}{\sqrt{n}}\Bigr )^u+\frac{x}{(24\alpha )^{\lceil \frac{w}{2}\rceil }w}\Bigl (\frac{1}{\sqrt{n}}\Bigr )^{w} \end{aligned}$$

and

$$\begin{aligned} g(w,x):=\frac{x}{(24\alpha )^{\lceil \frac{w}{2}\rceil }w}\Bigl (\frac{1}{\sqrt{n}}\Bigr )^{w}. \end{aligned}$$

Similarly, if we subtract from the upper bound for $m \rightarrow m-1$ in (6.3) the upper bound for *m*, we obtain $g(2m-2,2)-f(2m,2)$. Hence in order to prove (6.5), it suffices to prove

6.6 $$\begin{aligned} f(2m,-4)>g(2m-2,-4) \quad \text { and }\quad f(2m,2)<g(2m-2,2). \end{aligned}$$

Analogously, in order to prove that if (6.4) holds for all $m\ge 3$ and all $n\ge y\ge 1$, then (6.4) holds for $m-1$ and all $n\ge y$, it suffices to prove

6.7 $$\begin{aligned} f(2m-1,-7)>g(2m-3,-7) \quad \text { and } \quad f(2m-1,2)<g(2m-3,2). \end{aligned}$$

For proving (6.6) and (6.7), we shall prove

6.8 $$\begin{aligned} f(w,x_0(w))>g(w-2,x_0(w)) \text { with } x_0(w):=\Bigl \{\begin{array}{cc} -4, &{} \text { if } w \text { is even} \\ -7, &{} \text {if } w \text { is odd} \end{array} \end{aligned}$$

and

6.9 $$\begin{aligned} f(w,y_0)<g(w-2,y_0) \text { with } y_0>0. \end{aligned}$$

From Lemmas 5.7 and 5.8, we have

$$\begin{aligned} \frac{\ell _w}{(24\alpha )^{\lfloor \frac{w}{2}\rfloor }w}\le g_w \le \frac{u_w}{(24\alpha )^{\lfloor \frac{w}{2}\rfloor }w} \end{aligned}$$

with

$$\begin{aligned} \ell _w:=\left\{ \begin{array}{cc} -\mu _1, &{} \text {if } w \text { is odd}\\ -1, &{} \text {if } w \text { is even}\end{array}\right. \text { and } u_w:=\left\{ \begin{array}{cc} -\mu _2, &{} \text {if } w \text { is odd}\\ 2\nu , &{} \text {if } w \text { is even}\end{array}\right. , \end{aligned}$$

where $\mu _1$ and $\mu _2$ are as in Lemma 5.9 and $\nu$ as in Definition 6.1. Consequently,

$$\begin{aligned} f(w,x_0)&=\sum _{u=w-2}^{w-1}g_u\Bigl (\frac{1}{\sqrt{n}}\Bigr )^u+\frac{x_0}{(24\alpha )^{\lceil \frac{w}{2}\rceil }w}\Bigl (\frac{1}{\sqrt{n}}\Bigr )^w\\&\ge \frac{\ell _{w-2}}{(24\alpha )^{\lfloor \frac{w-2}{2}\rfloor }(w-2)}\Bigl (\frac{1}{\sqrt{n}}\Bigr )^{w-2}+\frac{\ell _{w-1}}{(24\alpha )^{\lfloor \frac{w-1}{2}\rfloor }(w-1)}\Bigl (\frac{1}{\sqrt{n}}\Bigr )^{w-1}\\&\quad +\frac{x_0}{(24\alpha )^{\lceil \frac{w}{2}\rceil }w}\Bigl (\frac{1}{\sqrt{n}}\Bigr )^w. \end{aligned}$$

In order to prove (6.8), it is enough to prove

6.10 $$\begin{aligned} \begin{aligned}&\frac{\ell _{w-2}}{w-2}+\frac{\ell _{w-1}}{(24\alpha )^{\alpha _w}(w-1)}\frac{1}{\sqrt{n}}+\frac{x_0}{(24\alpha )^{\beta _w}w}\frac{1}{n}>\frac{x_0}{(24\alpha )^{\delta _w}(w-2)}, \end{aligned} \end{aligned}$$

where

$$\begin{aligned} \begin{aligned}&\alpha _w=\Big \lfloor \frac{w-1}{2}\Big \rfloor -\Big \lfloor \frac{w-2}{2}\Big \rfloor =\Bigl \{\begin{array}{cc} 0, &{} \text { if } w \text { is even}\\ 1,&{} \text { if } w \text { is odd}\end{array},\\&\ \beta _w=\Big \lceil \frac{w}{2}\Big \rceil -\Big \lfloor \frac{w-2}{2}\Big \rfloor =\Bigl \{\begin{array}{cc} 1, &{} \text { if } w \text { is even}\\ 2,&{} \text { if } w \text { is odd}\end{array} ,\\&\text { and }\delta _w=\Big \lceil \frac{w-2}{2}\Big \rceil -\Big \lfloor \frac{w-2}{2}\Big \rfloor =\Bigl \{\begin{array}{cc} 0, &{} \text { if }w \text { is even}\\ 1,&{} \text { if }w\text { is odd}\end{array}. \end{aligned} \end{aligned}$$

Inequality (6.10) is equivalent to

$$\begin{aligned} \begin{aligned}&\Bigl (\ell _{w-2}-\frac{x_0}{(24\alpha )^{\alpha _w}}\Bigr )\frac{1}{w-2}>-\frac{\ell _{w-1}}{(24\alpha )^{\delta _w}(w-1)}\frac{1}{\sqrt{n}}+\frac{x_0}{(24\alpha )^{\beta _w}w}\frac{1}{n}, \end{aligned} \end{aligned}$$

which is implied by

6.11 $$\begin{aligned} \begin{aligned}&\Bigl (\ell _{w-2}-\frac{x_0}{(24\alpha )^{\alpha _w}}\Bigr )\frac{1}{w-2}>-\Bigl (\frac{\ell _{w-1}}{(24\alpha )^{\alpha _w}(w-1)}+\frac{x_0}{(24\alpha )^{\beta _w}w}\Bigr )\frac{1}{\sqrt{n}} \end{aligned} \end{aligned}$$

since $\delta _{w}=\alpha _{w}$, $x_0<0$ and $\frac{1}{\sqrt{n}}\ge \frac{1}{n}$ for all $n\ge 1$. Inequality (6.11) is equivalent to

$$\begin{aligned} \begin{aligned}&n\ge \Bigg \lceil \frac{(w-2)^2\Bigl (\frac{\ell _{w-1}}{(24\alpha )^{\alpha _w}(w-1)}+\frac{x_0}{(24\alpha )^{\beta _w}w}\Bigr )^2}{\Bigl (\ell _{w-2}-\frac{x_0}{(24\alpha )^{\alpha _w}}\Bigr )^2}\Bigg \rceil =:N_1(w,x_0). \end{aligned} \end{aligned}$$

We checked with Mathematica that $N_1(w,x_0(w))\le 1$; see the Appendix, Sect. 3.

Similarly to above, for $y_0>0$ one has,

$$\begin{aligned} \begin{aligned} f(w,y_0)&=\sum _{u=w-2}^{w-1}g_u\Bigl (\frac{1}{\sqrt{n}}\Bigr )^u+\frac{y_0}{(24\alpha )^{\lceil \frac{w}{2}\rceil }w}\Bigl (\frac{1}{\sqrt{n}}\Bigr )^w\\&\le \frac{u_{w-2}}{(24\alpha )^{\lceil \frac{w-2}{2}\rceil }(w-2)} \Bigl (\frac{1}{\sqrt{n}}\Bigr )^{w-2}+\frac{u_{w-1}}{(24\alpha )^{\lceil \frac{w-1}{2}\rceil }(w-1)} \Bigl (\frac{1}{\sqrt{n}}\Bigr )^{w-1}\\&\quad +\frac{y_0}{(24\alpha )^{\lceil \frac{w}{2}\rceil }w}\Bigl (\frac{1}{\sqrt{n}}\Bigr )^w. \end{aligned} \end{aligned}$$

In order to prove (6.9), it is enough to show

$$\begin{aligned} \begin{aligned}&\frac{u_{w-2}}{(24\alpha )^{\lceil \frac{w-2}{2}\rceil }(w-2)}\Bigl (\frac{1}{\sqrt{n}}\Bigr )^{w-2}+\frac{u_{w-1}}{(24\alpha )^{\lceil \frac{w-1}{2}\rceil }(w-1)}\Bigl (\frac{1}{\sqrt{n}}\Bigr )^{w-1}\\&\quad +\frac{y_0}{(24\alpha )^{\lceil \frac{w}{2}\rceil }w}\Bigl (\frac{1}{\sqrt{n}}\Bigr )^w<\frac{y_0}{(24\alpha )^{\lceil \frac{w-2}{2}\rceil }(w-2)}\Bigl (\frac{1}{\sqrt{n}}\Bigr )^{w-2}. \end{aligned} \end{aligned}$$

This last inequality can be rewritten as the following equivalent inequality,

$$\begin{aligned} \begin{aligned}&\frac{u_{w-2}}{w-2}+\frac{u_{w-1}}{(24\alpha )^{\alpha _w}(w-1)}\frac{1}{\sqrt{n}}+\frac{y_0}{(24\alpha )^{\beta _w}w}\frac{1}{n}<\frac{y_0}{(24\alpha )^{\alpha _w}(w-2)}, \end{aligned} \end{aligned}$$

which is implied by

6.12 $$\begin{aligned} \begin{aligned}&\Bigl (\frac{y_0}{(24\alpha )^{\alpha _w}}-u_{w-2}\Bigr )\frac{1}{w-2}>\Bigl (\frac{u_{w-1}}{(24\alpha )^{\alpha _w}(w-1)}+\frac{y_0}{(24\alpha )^{\beta _w}w}\Bigr )\frac{1}{\sqrt{n}} \end{aligned} \end{aligned}$$

since $y_0>0$ and $\frac{1}{\sqrt{n}}\ge \frac{1}{n}$. Inequality (6.12) is equivalent to

$$\begin{aligned} \begin{aligned}&n\ge \Bigg \lceil \frac{(w-2)^2\Bigl (\frac{u_{w-1}}{(24\alpha )^{\alpha _w}(w-1)}+\frac{y_0}{(24\alpha )^{\beta _w}w}\Bigr )^2}{\Bigl (\frac{y_0}{(24\alpha )^{\alpha _w}}-u_{w-2}\Bigr )^2}\Bigg \rceil =:N_2(w,y_0). \end{aligned} \end{aligned}$$

We checked using Mathematica that $N_2(w,y_0)\le 1$ for all $y_0\ge 1$; see the Appendix, Sect. 3.

We have checked with Mathematica that (6.3) holds for $m\in \{2,\dots ,10\}$ and $n\in {\mathbb {N}}$ such that

6.13 $$\begin{aligned} g(2m-2)< n\le g(2m). \end{aligned}$$

Now (6.3) is true for $m=10$ and $n>g(2m)$. Next, assume that (6.3) is true for $m=N$ with $2\le N\le 10$ and $n>g(2N)$. Then, as shown above, (6.3) is true for $m=N-1$ if $n>g(2N)$. By (6.13), (6.3) is true for $m=N-1$ if $g(2N-2)< n\le g(2N)$. This implies that (6.3) is true for $m=N-1$ and $n>g(2N-2)$. Hence the result follows inductively. The proof of (6.4) is done analogously. $\square$

Finally, we are put into the position to prove Theorem 1.1.

### Proof of Theorem 1.1

We apply (6.3) in Theorem 6.6, with $m=1$. Then for $n\ge 1$, we have

$$\begin{aligned} \begin{aligned}&-\log 4\sqrt{3}-\log n+\pi \sqrt{\frac{2n}{3}}-\sqrt{6}\Bigl (\frac{\pi }{144}+\frac{1}{2\pi }\Bigr )\frac{1}{\sqrt{n}}-\frac{2}{24\alpha }\frac{1}{n}\\&\quad<\log p(n)<-\log 4\sqrt{3}-\log n+\pi \sqrt{\frac{2n}{3}}-\sqrt{6}\Bigl (\frac{\pi }{144}+\frac{1}{2\pi }\Bigr )\frac{1}{\sqrt{n}}+\frac{1}{24\alpha }\frac{1}{n}. \end{aligned} \end{aligned}$$

Noting that $\sqrt{6}\Bigl (\frac{\pi }{144}+\frac{1}{2\pi }\Bigr )=0.44\dots$ finishes the proof. $\square$

## An application to Chen–DeSalvo–Pak log concavity result

In 2010 at FPSAC [3], William Chen conjectured that $\{p(n)\}_{n\ge 26}$ is log-concave and that for $n\ge 1$,

7.1 $$\begin{aligned} p(n)^2<\Bigl (1+\frac{1}{n}\Bigr )p(n-1)p(n+1). \end{aligned}$$

DeSalvo and Pak [6] proved these two conjectures. Moreover, they refined (7.1) by proposing the following conjecture:

7.2 $$\begin{aligned} p(n)^2<\Bigl (1+\frac{\pi }{\sqrt{24}n^{3/2}}\Bigr )p(n-1)p(n+1), \ n\ge 45. \end{aligned}$$

Chen, Wang, and Xie [2] gave an affirmative answer to (7.2). In this section, using Theorem 6.6, we continue this research by obtaining the following inequality:

$$\begin{aligned}&\Bigl (1+\frac{\pi }{\sqrt{24}n^{3/2}}-\frac{1}{n^2}\Bigr )p(n-1)p(n+1)\\&<p(n)^2<\Bigl (1+\frac{\pi }{\sqrt{24}n^{3/2}}\Bigr )p(n-1)p(n+1); \end{aligned}$$

for a more precise statement see Theorem 7.6. Note that the right inequality is just (7.2), but we give here our proof in order to show that, alternatively, one can obtain this from Theorem 6.6. In order to achieve our goal, we also need to prove the Lemmas 7.3 to 7.5 in this section. These lemmas deal with estimating the tail of an infinite series involving standard binomial coefficients.

### Proposition 7.1

For $s\ge 1$ and $k\ge 0$ we have

$$\begin{aligned} \left( {\begin{array}{c}-\frac{2s-1}{2}\\ k\end{array}}\right) =\frac{(-1)^k}{4^k}\frac{\left( {\begin{array}{c}2s+2k-2\\ s+k-1\end{array}}\right) \left( {\begin{array}{c}s+k-1\\ s-1\end{array}}\right) }{\left( {\begin{array}{c}2s-2\\ s-1\end{array}}\right) } \end{aligned}$$

and

$$\begin{aligned} \left( {\begin{array}{c}-s\\ k\end{array}}\right) =(-1)^k\left( {\begin{array}{c}s+k-1\\ s-1\end{array}}\right) . \end{aligned}$$

### Proof

By simplifying quotients formed by taking each expression in $k+1$ divided by the original expression in *k*. $\square$

### Lemma 7.2

For $$k,m\ge 0$$ and $$s\ge 1$$,

7.3 $$\begin{aligned} \left( {\begin{array}{c}s-1+m+k\\ s-1\end{array}}\right) \le \left( {\begin{array}{c}s-1+m\\ s-1\end{array}}\right) s^k. \end{aligned}$$

### Proof

From

$$\begin{aligned} \left( {\begin{array}{c}s-1+m+k\\ s-1\end{array}}\right) =\frac{(s-1+m+k)!}{(s-1)!(m+k)!}=\left( {\begin{array}{c}s-1+m\\ s-1\end{array}}\right) \frac{(s+m)\cdots (s+m+k-1)}{(m+1)\cdots (m+k)} \end{aligned}$$

we have $\frac{s+m+j}{m+j+1}\le s$ for each $0\le j \le k-1$; this is because

$$\begin{aligned} s+m+j\le s(m+j+1)\Leftrightarrow m(s-1)+j(s-1)\ge 0. \end{aligned}$$

This proves (7.3). $$\square $$

### Lemma 7.3

For $n,s\ge 1$, $m\ge 0$, and $n>2s$ let

$$\begin{aligned} b_{m,n}(s):=\frac{4\sqrt{s}}{\sqrt{s+m-1}}\left( {\begin{array}{c}s+m-1\\ s-1\end{array}}\right) \frac{1}{n^m}, \end{aligned}$$

then

7.4 $$\begin{aligned} -b_{m,n}(s)< \sum _{k=m}^{\infty }\left( {\begin{array}{c}-\frac{2s-1}{2}\\ k\end{array}}\right) \frac{1}{n^k}< b_{m,n}(s) \end{aligned}$$

and

7.5 $$\begin{aligned} 0< \sum _{k=m}^{\infty }\left( {\begin{array}{c}-\frac{2s-1}{2}\\ k\end{array}}\right) \frac{(-1)^k}{n^k}<b_{m,n}(s). \end{aligned}$$

### Proof

For $$s\ge 1$$:

$$\begin{aligned} \Bigl |\sum _{k=m}^{\infty }\left( {\begin{array}{c}-\frac{2s-1}{2}\\ k\end{array}}\right) \frac{1}{n^k}\Bigr |= & {} \Bigl |\sum _{k=m}^{\infty }\frac{(-1)^k}{4^k}\frac{\left( {\begin{array}{c}2s+2k-2\\ s+k-1\end{array}}\right) \left( {\begin{array}{c}s+k-1\\ s-1\end{array}}\right) }{\left( {\begin{array}{c}2s-2\\ s-1\end{array}}\right) }\frac{1}{n^k}\Bigr | \text { (by Proposition } \mathrm{7.1}\mathrm{)}\\\le & {} \sum _{k=m}^{\infty }\frac{1}{4^k}\frac{\left( {\begin{array}{c}2s+2k-2\\ s+k-1\end{array}}\right) \left( {\begin{array}{c}s+k-1\\ s-1\end{array}}\right) }{\left( {\begin{array}{c}2s-2\\ s-1\end{array}}\right) }\frac{1}{n^k}\\\le & {} \sum _{k=m}^{\infty }\frac{2\sqrt{s-1}}{\sqrt{\pi (s+k-1)}}\left( {\begin{array}{c}s+k-1\\ s-1\end{array}}\right) \frac{1}{n^k}\left( \text { using } \frac{4^n}{2\sqrt{n}}\le \left( {\begin{array}{c}2n\\ n\end{array}}\right) \le \frac{4^n}{\sqrt{\pi n}}\right) \\< & {} \frac{2\sqrt{s-1}}{\sqrt{s+m-1}}\sum _{k=m}^{\infty }\left( {\begin{array}{c}s-1+k\\ s-1\end{array}}\right) \frac{1}{n^k}\\&\times \left( \text {using } \frac{1}{\sqrt{\pi }}<1 \text { and } \frac{1}{\sqrt{s+k-1}}\le \frac{1}{\sqrt{s+m-1}}\text { for all } k\ge m \right) \\= & {} \frac{2\sqrt{s-1}}{\sqrt{s+m-1}}\sum _{k=0}^{\infty }\left( {\begin{array}{c}s-1+m+k\\ s-1\end{array}}\right) \frac{1}{n^{m+k}}\\= & {} \frac{2\sqrt{s-1}}{\sqrt{s+m-1}}\frac{1}{n^m}\sum _{k=0}^{\infty }\left( {\begin{array}{c}s-1+m+k\\ s-1\end{array}}\right) \frac{1}{n^{k}}. \end{aligned}$$

Now we apply Lemma 7.2 to obtain,

$$\begin{aligned} \begin{aligned} \Bigl |\sum _{k=m}^{\infty }\left( {\begin{array}{c}-\frac{2s-1}{2}\\ k\end{array}}\right) \frac{1}{n^k}\Bigr |&\le \frac{2\sqrt{s-1}}{\sqrt{s-1+m}}\frac{1}{n^m}\left( {\begin{array}{c}s-1+m\\ s-1\end{array}}\right) \sum _{k=0}^{\infty }\frac{s^k}{n^k}\\&=\frac{2\sqrt{s-1}}{\sqrt{s+m-1}}\left( {\begin{array}{c}s-1+m\\ s-1\end{array}}\right) \frac{1}{n^m}\frac{n}{n-s}<b_{m,n}(s), \end{aligned} \end{aligned}$$

where the latter inequality is by $n>2s$. This proves (7.4). Moreover, the bound we obtained also works for

$$\begin{aligned} \sum _{k=m}^{\infty }\frac{1}{4^k}\frac{\left( {\begin{array}{c}2s+2k-2\\ s+k-1\end{array}}\right) \left( {\begin{array}{c}s+k-1\\ s-1\end{array}}\right) }{\left( {\begin{array}{c}2s-2\\ s-1\end{array}}\right) }\frac{1}{n^k}, \end{aligned}$$

because this term showed up along the way in the proof of the previous case. Hence applying Proposition 7.1 implies (7.5). $\square$

### Lemma 7.4

For $n,s \ge 1$, $m\ge 0$, and $n>2s$ let

$$\begin{aligned} \beta _{m,n}(s):=\frac{2}{n^m}\left( {\begin{array}{c}s+m-1\\ s-1\end{array}}\right) , \end{aligned}$$

then

7.6 $$\begin{aligned} -\beta _{m,n}(s)<\sum _{k=m}^{\infty }\left( {\begin{array}{c}-s\\ k\end{array}}\right) \frac{1}{n^k}<\beta _{m,n}(s) \end{aligned}$$

and

7.7 $$\begin{aligned} 0<\sum _{k=m}^{\infty }\left( {\begin{array}{c}-s\\ k\end{array}}\right) \frac{(-1)^k}{n^k}<\beta _{m,n}(s). \end{aligned}$$

### Proof

$$\begin{aligned} \begin{aligned} \Bigl |\sum _{k=m}^{\infty }\left( {\begin{array}{c}-s\\ k\end{array}}\right) \frac{1}{n^k}\Bigr |&=\Bigl |\sum _{k=m}^{\infty }(-1)^k\left( {\begin{array}{c}s+k-1\\ s-1\end{array}}\right) \frac{1}{n^k}\Bigr | \ \text { (by Proposition } \mathrm{7.1}\mathrm{)}\\&\le \sum _{k=m}^{\infty }\left( {\begin{array}{c}s+k-1\\ s-1\end{array}}\right) \frac{1}{n^k}\\&=\frac{1}{n^m}\sum _{k=0}^{\infty }\left( {\begin{array}{c}s+k-1+m\\ s-1\end{array}}\right) \frac{1}{n^k}\\&<\frac{1}{n^m}\left( {\begin{array}{c}s-1+m\\ s-1\end{array}}\right) \sum _{k=0}^{\infty }\frac{s^k}{n^k} \ \text { (by Lemma }\mathrm{7.2}\mathrm{)}, \end{aligned} \end{aligned}$$

and geometric series summation implies (7.6). The proof of (7.7) is analogous. $\square$

Finally, we need another similar lemma which is easy to prove.

### Lemma 7.5

For $m, n, s\ge 1$ and $n>2s$ let

$$\begin{aligned} c_{m,n}(s):=\frac{2}{m}\frac{s^m}{n^m}. \end{aligned}$$

Then

$$\begin{aligned} -c_{m,n}(s)<\sum _{k=m}^{\infty }\frac{(-1)^{k+1}}{k}\frac{s^k}{n^k}<c_{m,n}(s) \quad \text { and } \quad -c_{m,n}(s)<-\sum _{k=m}^{\infty }\frac{1}{k}\frac{s^k}{n^k}<0 \end{aligned}$$

and

7.8 $$\begin{aligned}&-\frac{c_{m,n}(s)}{\sqrt{m}}<\sum _{k=m}^{\infty }\left( {\begin{array}{c}1/2\\ k\end{array}}\right) \frac{s^k}{n^k}<\frac{c_{m,n}(s)}{\sqrt{m}} \quad \text { and } \quad \nonumber \\&-\frac{c_{m,n}(s)}{\sqrt{m}}<\sum _{k=m}^{\infty }\left( {\begin{array}{c}1/2\\ k\end{array}}\right) \frac{(-1)^ks^k}{n^k}<0. \end{aligned}$$

The following theorem was announced in the abstract; its proof is the goal of this section. To arrive at the intermediate inequality (7.13), we need our main result, Theorem 6.6. For the remainder of the proof, one spends some time on simplifying (7.13) in order to arrive at the desired form. In order to do, one needs the Lemmas 7.3 to 7.5 which we have proven above in this section.

### Theorem 7.6

For $$n\ge 45$$,

$$\begin{aligned} p(n)^2<\Bigl (1+\frac{\pi }{\sqrt{24}n^{3/2}}\Bigr )p(n-1)p(n+1), \end{aligned}$$

and for $$n\ge 120$$

$$\begin{aligned} p(n)^2>\Bigl (1+\frac{\pi }{\sqrt{24}n^{3/2}}-\frac{1}{n^2}\Bigr )p(n-1)p(n+1). \end{aligned}$$

### Proof

We set $m=3$ in the first equation of Theorem 6.6, which gives for all $n\ge \Bigl \lceil g(6) \Bigr \rceil$ that

$$\begin{aligned} \begin{aligned}&\underbrace{P_n(5)-\frac{2}{3(24\alpha )^3}\frac{1}{n^3}}_{=:l(n)}<\log p(n)<\underbrace{P_n(5)+\frac{1}{3(24\alpha )^3}\frac{1}{n^3}}_{=:u(n)}, \end{aligned} \end{aligned}$$

using the notation from Definition 6.4. This inequality has the form

7.9 $$\begin{aligned} l(n)<\log p(n) < u(n). \end{aligned}$$

By substituting *n* by $n+1$ and multiplying by $-1$ into (7.9) we obtain

7.10 $$\begin{aligned} -u(n+1)<-\log p(n+1)< -l(n+1), \end{aligned}$$

and by substituting *n* by $n-1$ and multiplying by $-1$ again into (7.9) gives

7.11 $$\begin{aligned} -u(n-1)<-\log p(n-1)<-l(n-1). \end{aligned}$$

Multiplying (7.9) by 2, and by adding (7.10) and (7.11), results in

7.12 $$\begin{aligned}&2l(n)-u(n-1)-u(n+1)<2\log p(n)-\log p(n-1)-\log p(n+1)\nonumber \\&\quad < 2u(n)-l(n-1)-l(n+1). \end{aligned}$$

We define

$$\begin{aligned} A_{1}(n):= & {} \log \left( 1+\frac{1}{n}\right) +\log \left( 1-\frac{1}{n}\right) ,\\ A_2(n):= & {} -\pi \sqrt{\frac{2n}{3}}\left( \sum _{k=1}^{\infty }\left( {\begin{array}{c}1/2\\ k\end{array}}\right) \frac{(-1)^k}{n^k}+\sum _{k=1}^{\infty }\left( {\begin{array}{c}1/2\\ k\end{array}}\right) \frac{1}{n^k}\right) , \end{aligned}$$

and for $$t\ge 3$$

$$\begin{aligned} A_t(n):=-\frac{g_{t-2}}{(\sqrt{n})^{t-2}}\left( \sum _{k=1}^{\infty }\left( {\begin{array}{c}-\frac{t-2}{2}\\ k\end{array}}\right) \frac{(-1)^k}{n^k}+\sum _{k=1}^{\infty }\left( {\begin{array}{c}-\frac{t-2}{2}\\ k\end{array}}\right) \frac{1}{n^k}\right) , \end{aligned}$$

where $g_n$ is as in Definition 5.1. Then from (7.12), by substituting *l*(*n*) and *u*(*n*) according to their definitions, we obtain

$$\begin{aligned}&-\frac{7}{(24\alpha )^3\cdot 3}\frac{1}{n^3}+\sum _{t=1}^7 A_t(n)<2 \log p(n)-\log p(n-1)-\log p(n+1)\\&\qquad \qquad \quad <\sum _{t=1}^7A_t(n)+\frac{8}{(24\alpha )^3}\frac{1}{n^3}, \end{aligned}$$

which implies

7.13 $$\begin{aligned}&-\frac{3}{(24\alpha )^3}\frac{1}{n^3}+\sum _{t=1}^7 A_t(n)<2 \log p(n)-\log p(n-1)-\log p(n+1)\nonumber \\&\qquad \qquad \quad <\sum _{t=1}^7A_t(n)+\frac{3}{(24\alpha )^3}\frac{1}{n^3}. \end{aligned}$$

Finally, we establish bounds for the $A_t(n)$. For $t=1$,

$$\begin{aligned} A_1(n)=\log \left( 1+\frac{1}{n}\right) +\log \left( 1-\frac{1}{n}\right) =-\frac{1}{n^2}-\frac{1}{2n^4}+\sum _{k=5}^{\infty }\frac{(-1)^{k+1}}{kn^k}-\sum _{k=5}^{\infty }\frac{1}{kn^k}. \end{aligned}$$

Taking $s=1$ and $m=5$ in Lemma 7.5 we have

$$\begin{aligned} -\frac{1}{n^2}-\frac{1}{2n^4}-\frac{4}{5n^5}<A_1(n)<-\frac{1}{n^2}-\frac{1}{2n^4}+\frac{2}{5n^5} \end{aligned}$$

which implies

7.14 $$\begin{aligned} -\frac{1}{n^2}-\frac{2}{n^3}<A_1(n)<-\frac{1}{n^2}. \end{aligned}$$

For $t=2$, note that

$$\begin{aligned} \begin{aligned} A_2(n)=&-\pi \sqrt{\frac{2n}{3}}\Bigl (-\frac{5}{64n^4}-\frac{1}{4n^3}+\sum _{k=5}^{\infty }\left( {\begin{array}{c}1/2\\ k\end{array}}\right) \frac{(-1)^k}{n^k}+\sum _{k=5}^{\infty }\left( {\begin{array}{c}1/2\\ k\end{array}}\right) \frac{1}{n^k}\Bigr ). \end{aligned} \end{aligned}$$

Applying Lemma 7.5, (7.8), with $s=1$ and $m=5$ gives

$$\begin{aligned}&-\pi \sqrt{\frac{2n}{3}}\Bigl (-\frac{1}{4n^2}-\frac{5}{64n^4}-\frac{4}{5\sqrt{5}}\frac{1}{n^5}\Bigr )\\&<A_2(n)<-\pi \sqrt{\frac{2n}{3}}\Bigl (-\frac{1}{4n^2}-\frac{5}{64n^4}+\frac{2}{5\sqrt{5}}\frac{1}{n^5}\Bigr ), \end{aligned}$$

which implies

7.15 $$\begin{aligned} \frac{\pi }{\sqrt{24}n^{3/2}}<A_2(n)<\frac{\pi }{\sqrt{24}n^{3/2}}+\frac{2}{n^{5/2}}. \end{aligned}$$

Next we consider odd indices; i.e., for $1\le t\le 3$,

$$\begin{aligned} \begin{aligned} A_{2t+1}(n)&=-\frac{g_{2t-1}}{(\sqrt{n})^{2t-1}}\Bigl (\frac{\Bigl (\frac{2t-1}{2}\Bigr )_2}{n^2}\\&\quad +\frac{\Bigl (\frac{2t-1}{2}\Bigr )_4}{12n^4}+\sum _{k=5}^{\infty }\left( {\begin{array}{c}-\frac{2t-1}{2}\\ k\end{array}}\right) \frac{(-1)^k}{n^k}+\sum _{k=5}^{\infty }\left( {\begin{array}{c}-\frac{2t-1}{2}\\ k\end{array}}\right) \frac{1}{n^k}\Bigr ), \end{aligned} \end{aligned}$$

where $(a)_k:=a(a-1)\dots (a-k+1)$. Applying Lemma 7.3 with $s=t$ and $m=5$ gives

$$\begin{aligned} \begin{aligned}&-\frac{g_{2t-1}}{(\sqrt{n})^{2t-1}}\Bigl (\frac{\Bigl (\frac{2t-1}{2}\Bigr )_2}{n^2}+\frac{\Bigl (\frac{2t-1}{2}\Bigr )_4}{12n^4}-\frac{4\sqrt{t}}{\sqrt{t+4}}\left( {\begin{array}{c}t+4\\ t-1\end{array}}\right) \frac{1}{n^5}\Bigr )<A_{2t+1}(n)\\&\quad <-\frac{g_{2t-1}}{(\sqrt{n})^{2t-1}}\Bigl (\frac{\Bigl (\frac{2t-1}{2}\Bigr )_2}{n^2}+\frac{\Bigl (\frac{2t-1}{2}\Bigr )_4}{12n^4}+\frac{8\sqrt{t}}{\sqrt{t+4}}\left( {\begin{array}{c}t+4\\ t-1\end{array}}\right) \frac{1}{n^5}\Bigr ), \end{aligned} \end{aligned}$$

which implies

7.16 $$\begin{aligned}&-\frac{3g_1}{4n^{5/2}}+\frac{4g_1}{\sqrt{5}}\frac{1}{n^3}<A_3(n)<-\frac{5g_1}{n^{5/2}}, \end{aligned}$$

7.17 $$\begin{aligned}&\frac{4\sqrt{6}g_3}{n^3}<A_5(n)<-\frac{29g_3}{n^{5/2}}, \end{aligned}$$

7.18 $$\begin{aligned}&\frac{4\sqrt{2}}{\sqrt{7}}\left( {\begin{array}{c}7\\ 2\end{array}}\right) \frac{g_5}{n^3}<A_7(n)<-\frac{117g_5}{n^{5/2}}. \end{aligned}$$

Finally, we consider even indices; i.e., for $1\le t\le 2$,

$$\begin{aligned} \begin{aligned} A_{2t+2}(n)&=-\frac{g_{2t}}{(\sqrt{n})^{2t}}\Bigl (\frac{\Bigl (-\frac{2t}{2}\Bigr )_2}{n^2}+\frac{\Bigl (-\frac{2t}{2}\Bigr )_4}{12n^4}\\&\quad +\sum _{k=5}^{\infty }\left( {\begin{array}{c}-\frac{2t}{2}\\ k\end{array}}\right) \frac{(-1)^k}{n^k}+\sum _{k=5}^{\infty }\left( {\begin{array}{c}-\frac{2t}{2}\\ k\end{array}}\right) \frac{1}{n^k}\Bigr ). \end{aligned} \end{aligned}$$

Applying Lemma 7.4 with $s=t$ and $m=5$, we obtain

$$\begin{aligned} \begin{aligned}&-\Bigl (\frac{(-t)_2}{n^2}+\frac{(-t)_4}{12n^4}-\frac{2}{n^5}\left( {\begin{array}{c}t+4\\ t-1\end{array}}\right) \Bigr )\frac{g_{2t}}{(\sqrt{n})^{2t}}<A_{2t+2}(n)\\&\quad <-\Bigl (\frac{(-t)_2}{n^2}+\frac{(-t)_4}{12n^4}+\frac{4}{n^5}\left( {\begin{array}{c}t+4\\ t-1\end{array}}\right) \Bigr )\frac{g_{2t}}{(\sqrt{n})^{2t}}. \end{aligned} \end{aligned}$$

From this,

7.19 $$\begin{aligned}&\frac{2g_2}{n^3}<A_4(n)<-\frac{8g_2}{n^{5/2}}, \end{aligned}$$

7.20 $$\begin{aligned}&\frac{12g_4}{n^3}<A_6(n)<-\frac{40g_4}{n^{5/2}}. \end{aligned}$$

Now, substituting (7.14) to (7.20) into (7.13) gives,

$$\begin{aligned} L(n)<2\log p(n)-\log p(n-1)-\log p(n+1)<U(n), \end{aligned}$$

where

$$\begin{aligned} L(n):= & {} \frac{\pi }{\sqrt{24}}\frac{1}{n^{3/2}}-\frac{1}{n^2}-\frac{3g_1}{4}\frac{1}{n^{5/2}}+\Bigl (-2+\frac{4g_1}{\sqrt{5}}+2g_2+4\sqrt{6}g_3+12g_4\\&+\frac{4\sqrt{2}}{\sqrt{7}}\left( {\begin{array}{c}7\\ 2\end{array}}\right) g_5-\frac{3}{(24\alpha )^3}\Bigr )\frac{1}{n^3} \end{aligned}$$

and

$$\begin{aligned} U(n):=\frac{\pi }{\sqrt{24}}\frac{1}{n^{3/2}}-\frac{1}{n^2}+\Bigl (2-5g_1-8g_2-29g_3-40g_4-117g_5+\frac{3}{(24\alpha )^3}\Bigr )\frac{1}{n^{5/2}}. \end{aligned}$$

By using numerical estimations of the coefficient of $1/n^{5/2}$ and of the coefficient of $1/n^3$ in the lower bound, and of the coefficient of $1/n^{5/2}$ in the upper bound above, we are led to

$$\begin{aligned} L_1(n)<2\log p(n)-\log p(n-1)-\log p(n+1)<U_1(n), \end{aligned}$$

with

$$\begin{aligned} L_1(n):=\frac{\pi }{\sqrt{24}}\frac{1}{n^{3/2}}-\frac{1}{n^2}+\frac{1}{4}\frac{1}{n^{5/2}}-\frac{4}{n^3} \; \text { and }\; U_1(n):=\frac{\pi }{\sqrt{24}}\frac{1}{n^{3/2}}-\frac{1}{n^2}+\frac{7}{n^{5/2}}. \end{aligned}$$

Next we observe that

$$\begin{aligned} -\frac{1}{n^2}+\frac{7}{n^{5/2}}<-\frac{\pi ^2}{48n^3} \quad \text { for all } n\ge 50 \end{aligned}$$

and

$$\begin{aligned} -\frac{1}{n^2}+\frac{\pi }{\sqrt{24}}\frac{1}{n^{3/2}}+\frac{1}{4}\frac{1}{n^{5/2}}-\frac{4}{n^3}>-\frac{1}{n^2}+\frac{\pi }{\sqrt{24}}\frac{1}{n^{3/2}} \text { for all } n\ge 257. \end{aligned}$$

Therefore, for $$n\ge 257$$,

7.21 $$\begin{aligned}&\frac{\pi }{\sqrt{24}n^{3/2}}-\frac{1}{n^2}<2\log p(n)-\log p(n-1)-\log p(n+1)\nonumber \\&<\frac{\pi }{\sqrt{24}n^{3/2}}-\frac{\pi ^2}{48n^3}. \end{aligned}$$

Because of $\log (1+x)<x$ for $x>0$, we have

7.22 $$\begin{aligned} \log \Bigl (1+\frac{\pi }{\sqrt{24}n^{3/2}}-\frac{1}{n^2}\Bigr )<\frac{\pi }{\sqrt{24}n^{3/2}}-\frac{1}{n^2}, \end{aligned}$$

and because of $x-\frac{x^2}{2}<\log (1+x)$ for all $x>0$, we have

7.23 $$\begin{aligned} \frac{\pi }{\sqrt{24}n^{3/2}}-\frac{\pi ^2}{48n^3}<\log \Bigl (1+\frac{\pi }{\sqrt{24}n^{3/2}}\Bigr ). \end{aligned}$$

Applying (7.22) and (7.23) to (7.21) gives

$$\begin{aligned}&\log \Bigl (1+\frac{\pi }{\sqrt{24}n^{3/2}}-\frac{1}{n^2}\Bigr )\\&<2\log p(n)-\log p(n-1)-\log p(n+1)\\&<\log \Bigl (1+\frac{\pi }{\sqrt{24}n^{3/2}}\Bigr ), \end{aligned}$$

which after exponentiation gives the desired result for $n\ge 257$. To extend the proofs of the statements for $n\ge 45$, resp. $n\ge 120$, is done by straight-forward numerics. $\square$

## Figures and Tables

**Fig. 3:**
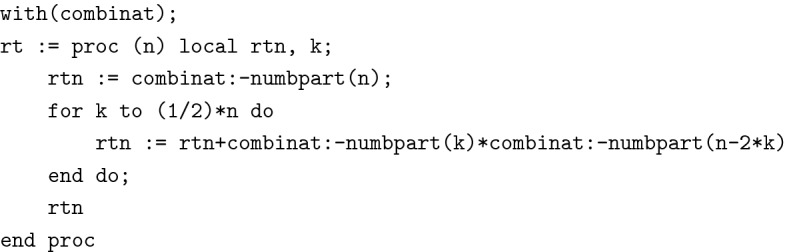
Procedure for computing the number of cubic partitions of *n*

**Fig. 4:**
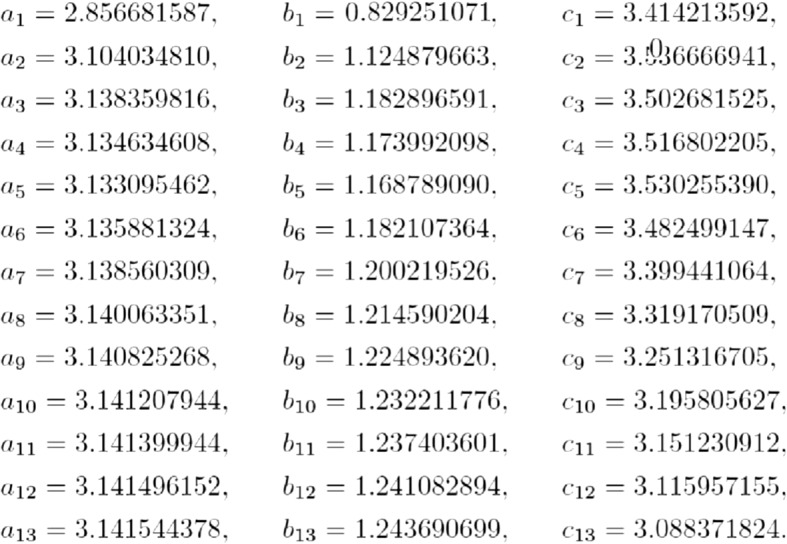
Numerical values of the $(a_k,b_k,c_k)$

## Appendix A

### A. 1 Methods to discover the results

We will describe very briefly the mathematical experiments used in this research. We want to point out that without these experiments, the theoretical results of this paper would never have been found. For this reason we feel that it is important to give at least a brief sketch of what led us to the final formulas and how we were led to conjecture special cases of related asymptotics. The final asymptotic formulas can easily be derived from our main result, Theorem 6.6 presented in Sect. 6.

In Sect. 3 we proved the inequality

7.24 $$\begin{aligned} \frac{e^{\pi \sqrt{\frac{2n}{3}}}}{4\sqrt{3}n} \Bigl (1 - \frac{1}{2\sqrt{n}}\Bigr )< p(n) < \frac{e^{\pi \sqrt{\frac{2n}{3}}}}{4\sqrt{3}n} \Bigl (1 - \frac{1}{3\sqrt{n}}\Bigr ), \end{aligned}$$

which was found by mathematical experiments. Our proof uses methods similar to those used in [6] and [1]. In our attempt to prove the following formula for the asymptotics of $\log p(n)$,

7.25 $$\begin{aligned} \log p(n) \sim \pi \sqrt{\frac{2n}{3}} - \log n - \log (4\sqrt{3}) - \frac{0.44\dots }{\sqrt{n}}, \end{aligned}$$

we first tried to prove the log-version of (7.24). However, we soon realized that this inequality is not sharp enough in order to prove (7.25). We noted that the inequality for *p*(*n*) in [1, Lemma 2.2] can be used instead. This formula says that for $n\ge 1206$,

7.26 $$\begin{aligned}&\frac{\sqrt{12}e^{\mu (n)}}{24n-1}\Bigl (1-\frac{1}{\mu (n)}-\frac{1}{\mu (n)^{10}}\Bigr )\nonumber \\&<p(n)<\frac{\sqrt{12}e^{\mu (n)}}{24n-1}\Bigl (1-\frac{1}{\mu (n)}+\frac{1}{\mu (n)^{10}}\Bigr ), \end{aligned}$$

where $\mu (n):=\frac{\pi }{6}\sqrt{24n-1}$. We observed that after taking the log of both sides, with some extra work, (7.25) can be proven. When we saw the asymptotics (1.3), discovered by Schoenfield and Kotesovec, we naturally wondered whether these asymptotics can also be proven by taking the $\log$ of an appropriate inequality. We observed that (7.26) is enough also to prove these asymptotics, and we observed that (7.26) can be used to prove an even more refined asymptotic formula that takes the form

$$\begin{aligned} \log p(n)\sim \pi \sqrt{\frac{2n}{3}}-\log n- \log 4\sqrt{3}+b_1 \Bigl (\frac{1}{\sqrt{n}}\Bigr )+b_2\Bigl (\frac{1}{\sqrt{n}}\Bigr )^2+\dots +b_9\Bigl (\frac{1}{\sqrt{n}}\Bigr )^9, \end{aligned}$$

where

$$\begin{aligned} b_1= & {} -\frac{\pi \sqrt{6}}{2^43^2}-\frac{\sqrt{6}}{2\pi }\approx -0.44328...,\\ b_2= & {} \frac{1}{3\cdot 2^3}-\frac{3}{2^2\pi ^2}\approx -0.034324...,\\ b_3= & {} -\frac{\pi \sqrt{6}}{2^93^3}-\frac{\sqrt{6}}{2^53\pi }-\frac{\sqrt{6}}{2^2\pi ^3}\approx -0.028428...,\\ b_4= & {} \frac{1}{2^73^2}-\frac{1}{2^5\pi ^2}-\frac{9}{2^4\pi ^4}\approx -0.0080728...,\\ b_5= & {} -\frac{\pi \sqrt{6}}{2^{13}3^4}-\frac{\sqrt{6}}{2^{10}3\pi }-\frac{\sqrt{6}}{2^6\pi ^3}-\frac{9\sqrt{6}}{5\cdot 8 \pi ^5}\approx -0.0033007...,\\ b_6= & {} \frac{1}{2^93^4}-\frac{1}{2^83\pi ^2}-\frac{3}{2^6\pi ^4}-\frac{9}{2^4\pi ^6}\approx -0.001174124716...,\\ b_7= & {} -\frac{5\pi \sqrt{6}}{2^{19}3^5}-\frac{5\sqrt{6}}{2^{14}3^3\pi }-\frac{5\sqrt{6}}{2^{11}3\pi ^3}-\frac{3\sqrt{6}}{2^7\pi ^5}-\frac{3^3\sqrt{6}}{2^47\pi ^7}\approx -0.00045651...,\\ b_8= & {} \frac{1}{2^{14}3^4}-\frac{1}{2^{11}3^2\pi ^2}-\frac{3}{2^{10}\pi ^4}-\frac{3^22}{2^7\pi ^6}-\frac{3^4}{2^7\pi ^8}\approx -0.00017464...,\\ b_9= & {} -\frac{7\pi \sqrt{6}}{2^{23}3^6}-\frac{35\sqrt{6}}{2^{20}3^4\pi }-\frac{35\sqrt{6}}{2^{15}3^3\pi ^3}-\frac{7\sqrt{6}}{2^{12}\pi ^5}-\frac{9\sqrt{6}}{2^8\pi ^7}-\frac{9\sqrt{6}}{2^5\pi ^9}\approx -0.000068757...,\\&\vdots \end{aligned}$$

Of course we wondered whether one can get an even better formula. The only obstacle that seemed to limit us was the 10 in the formula (7.26) above. This led us to look into the details of the proof of (7.26), and we observed that the 10 can be replaced by a *k*. This then led us to the discovery of the complete asymptotics. That is, we also got $b_{10}, b_{11},\dots$, etc. At this point we still were not fully satisfied. Even though we observed that the formula (7.26) could be generalized, it was not a proper generalization because we could not say explicitly for which precise range of *n* the generalized inequality (4.3) for *p*(*n*) holds. We only could say that there is some sufficiently big constant *C*(*k*) such that (4.3) for all $n>C(k)$.

We felt that this is not a proper generalization because (7.26) gives *C*(10) explicitly, namely $C(10)=1206$. After some work, we realized that we can obtain an explicit expression for *C*(*k*), which is very close to the optimal value, according to mathematical experiments. This *C*(*k*) is our *g*(*k*) of Sect. 4 where we gave a generalization of (7.26).

Because (7.26) could be generalized, we suspected that also (7.24) could be generalized. The difference between the two inequalities is that (7.24) is in terms of $\sqrt{n}$, while (7.26) is in terms of $\mu (n)$. We again took the $\log$ of both sides of the generalized version of (7.26) and aimed not only at getting a refined asymptotic but rather a new type of inequality. This was achieved in Sect. 6. However, even after we found a preliminary version of Theorem 6.6, still something was missing. We wondered whether we can guarantee that this inequality is optimal in some sense, and not overestimated. After various experiments, we got control in the form (6.3) and (6.4), where the error term in the inequality cannot be improved to a smaller integer in the numerator—the same time keeping the statement unaltered. This is the point where we stopped.

### A. 2 Discovery of Kotesovec’s formula (1.5) by regression analysis

We used the procedure shown in Fig. 3 to compute the sequence *a*(*n*) defined in (1.4). This procedure works fine for computing *a*(*n*) in the range $1\le n\le 2^{15}$. The computation took 24 h on a notebook computer with Intel Core i7 CPU.

To find the approximate relation between $\log a(n),\sqrt{n}$, and $\log (n)$, substitute the values $n = 2^k,\;2^{k + 1},\;2^{k +2}$ into the target expression,

$$\begin{aligned} \log a(n) \sim \alpha \cdot \sqrt{n} - \beta \cdot \log (n) - \log (\gamma ), \end{aligned}$$

to obtain a system with three equations:

$$\begin{aligned} \left\{ {{\begin{array}{*{20}c} {\log _2 a(2^k) = a_k \log _2 (e) \cdot \sqrt{2^k} - b_k \cdot k - c_k + \varepsilon _k ,\quad \quad \quad \quad } \\ {\log _2 a(2^{k + 1}) = a_k \log _2 (e) \cdot \sqrt{2^{k + 1}} - b_k \cdot (k + 1) - c_k + \varepsilon _{k + 1} ,\;} \\ {\log _2 a(2^{k + 2}) = a_k \log _2 (e) \cdot \sqrt{2^{k + 2}} - b_k \cdot (k + 2) - c_k + \varepsilon _{k + 2} ,} \\ \end{array} }} \right. \end{aligned}$$

and solve it successively for *k* from 1 to 13. Let $(a_k ,b_k ,c_k)$ be the solution of the above equation system under the assumption $\varepsilon _k = \varepsilon _{k + 1} = \varepsilon _{k +2} = 0$ for all $k\in \{1,\ldots ,13\}$. The numerical values of the $(a_k,b_k,c_k)$ are presented in Fig. 4. In the limit $k\rightarrow \infty$,

$$\begin{aligned} \begin{array}{l} a_k = \frac{\log _2 a(2^k) + \log _2 a(2^{k + 2}) - 2\log _2 a(2^{k + 1})}{(3 - 2\sqrt{2} )\sqrt{2^k} \log _2 (e)} \rightarrow \alpha , \\ b_k = \frac{\log _2 a(2^k) + \log _2 a(2^{k + 2}) - 2\log _2 a(2^{k + 1})}{\sqrt{2} - 1} - \{\log _2 a(2^{k + 1}) - \log _2 a(2^k)\} \rightarrow \beta , \\ c_k = \frac{2^{a_k \log _2 (e)\sqrt{2^k} - k\;b_k }}{a(2^k)} \rightarrow \log _2 (\gamma ) . \\ \end{array} \end{aligned}$$

The numerical values in Fig. 4 clearly support the precise values

$$\begin{aligned} \alpha = \pi ,\quad \beta = \frac{5}{4},\quad \gamma = 2^3 = 8. \end{aligned}$$

Note that we have used a sub-sequence $a(2^k)$, $k = 1,2, \ldots , 15$. The regression analysis to obtain the numerical data for Figs. 1 and 2 are rather routine, so we will not list any further details here.

### A. 3 Mathematica computations used in the proof of Theorem 6.6

We present Mathematica computations needed in the proof of Theorem 6.6. Note that in order to complete the proof of Theorem 6.6 we needed to bound four terms by 1; however, in each inequality proven with Mathematica as shown below, we checked that each inequality holds in fact for bounds smaller than 1, namely $\frac{1}{5}$, $\frac{1}{3}$, $\frac{1}{26}$, and $\frac{1}{26}$. The Mathematica computations are based on Cylindrical Algebraic Decomposition [5].

## Footnotes

1 In the literature, the Hardy–Ramanujan–Rademacher is also called an asymptotic formula/approximation. However, it is built by an expression of substantially more complicated type. For example, the log concavity of *p*(*n*) follows nontrivially from it, as shown in the work of DeSalvo and Pak [6].

2 The fourth author of this paper told the result to V. Kotesovec in May 2016 and got a reply in January 2017 that the precise value of *C* could be Pi/16+15/(8*Pi)=0.7931...

3 Note that in [1] *R*(*n*, *N*) is denoted by $R_2(n,N)$.
